# The Head and Neck Anatomy of Sea Turtles (Cryptodira: Chelonioidea) and Skull Shape in Testudines

**DOI:** 10.1371/journal.pone.0047852

**Published:** 2012-11-07

**Authors:** Marc E. H. Jones, Ingmar Werneburg, Neil Curtis, Rod Penrose, Paul O’Higgins, Michael J. Fagan, Susan E. Evans

**Affiliations:** 1 Research Department of Cell and Developmental Biology, UCL, University College London, London, England, United Kingdom; 2 Eberhard Karls Universität, Fachbereich Geowissenschaften, Tübingen, Germany; 3 RIKEN Center for Developmental Biology, Chuo-ku, Kobe, Japan; 4 Paläontologisches Institut und Museum der Universität Zürich, Zürich, Switzerland; 5 Department of Engineering, University of Hull, Hull, England, United Kingdom; 6 United Kingdom Cetacean Strandings Investigation Programme, Marine Environmental Monitoring, Penwalk, Llechryd, Caredigion, Cymru, United Kingdom; 7 Centre for Anatomical and Human Sciences, The Hull York Medical School, University of York, York, England, United Kingdom; Raymond M. Alf Museum of Paleontology, United States of America

## Abstract

**Background:**

Sea turtles (Chelonoidea) are a charismatic group of marine reptiles that occupy a range of important ecological roles. However, the diversity and evolution of their feeding anatomy remain incompletely known.

**Methodology/Principal Findings:**

Using computed tomography and classical comparative anatomy we describe the cranial anatomy in two sea turtles, the loggerhead (*Caretta caretta*) and Kemp’s ridley (*Lepidochelys kempii*), for a better understanding of sea turtle functional anatomy and morphological variation. In both taxa the temporal region of the skull is enclosed by bone and the jaw joint structure and muscle arrangement indicate that palinal jaw movement is possible. The tongue is relatively small, and the hyoid apparatus is not as conspicuous as in some freshwater aquatic turtles. We find several similarities between the muscles of *C*. *caretta* and *L*. *kempii*, but comparison with other turtles suggests only one of these characters may be derived: connection of the m. adductor mandibulae internus into the Pars intramandibularis via the Zwischensehne. The large fleshy origin of the m. adductor mandibulae externus Pars superficialis from the jugal seems to be a characteristic feature of sea turtles.

**Conclusions/Significance:**

In *C*. *caretta* and *L*. *kempii* the ability to suction feed does not seem to be as well developed as that found in some freshwater aquatic turtles. Instead both have skulls suited to forceful biting. This is consistent with the observation that both taxa tend to feed on relatively slow moving but sometimes armoured prey. The broad fleshy origin of the m. adductor mandibulae externus Pars superficialis may be linked to thecheek region being almost fully enclosed in bone but the relationship is complex.

## Introduction

**Figure 1:**
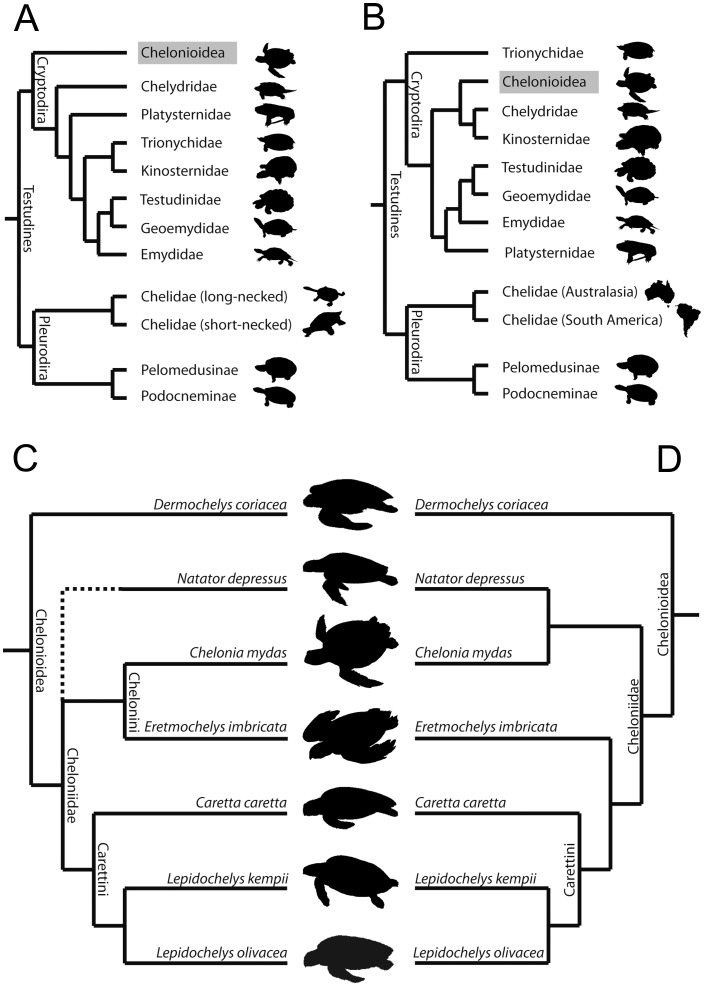
Phylogenetic relationships of sea turtles (Chelonioidea). The alternative positions of Chelonioidea within Cryptodira according to (**A**) Joyce [40] and (**B**) Shaffer [41]. Alternative relationships of chelonioid species following the hypotheses of (**C)** Gaffney and Meylan [202] (*Natator depressus* was not included in that analysis) and (**D**) Thomson and Shaffer [43]. For further details see Werneburg [44]. Names of higher taxa follow Gaffney and Meylan [202]. Silhouette images not to scale.

Sea turtles (Chelonioidea) are adapted to a marine lifestyle in possessing flippers, hydrodynamic shells, lacrimal salt glands, and a specialised physiology for diving [1]–[18]. Their fossil record is considered by some to extends at least as far back as the Early Cretaceous, 110 million years ago (e.g. [9], [19]–[23]), and the group contains seven living species: *Caretta caretta*, *Chelonia mydas*, *Dermochelys coriacea*, *Eretmochelys imbricata*, *Lepidochelys kempii*, *Lepidochelys olivacea*, and *Natator depressus* (Fig. 1) (*sensu* Fritz and Havaš [24]). These turtles pursue a range of feeding strategies [2], [23], [25].

Extant sea turtles are of particular interest to comparative anatomists and evolutionary biologists because these turtles possess a temporal region largely enclosed by bone. There are no fenestrations, the emarginations are small, and the adductor chamber is almost entirely enclosed by dermatocranial bones [26]–[29]. This contrasts with the condition found in many other turtles, whose skulls can show dramatic emarginations and are often more lightly built [29]–[32]. Historically, some authors considered the sea turtle phenotype to resemble or even represent the ancestral condition of Testudines [26], [33]–[35]. However, early fossil Chelonioidea (e.g. *Toxochelys latiremis*, *Allopleuron*, [22], [29], [36], [37]) possess skulls with an emarginated temporal region comparable to that of many extant non-chelonioid taxa. In addition, although the exact phylogenetic position of Chelonioidea is still uncertain recent phylogenetic analyses nest chelonioids within Cryptodira amongst clades of turtles with emarginated skulls [37]–[44]. Therefore, as proposed by Boulenger [45], Goodrich ([46]: 273, [47]: 352–354), and others subsequently, the condition in sea turtles is secondary. As the shapes of skull bones are highly influenced by loading from loads experienced during feeding (e.g. [48]–[56]), this raises questions as to whether the sea turtle skull with its enclosed termporal region is associated with a particular muscle architecture. Unfortunately, the cranial muscles of most sea turtles have not been fully surveyed, and previous descriptions are complicated by inconsistent homology or absence of detail (e.g. [57]).

Here, we describe the head and neck anatomy of *Caretta caretta* and *Lepidochelys kempii*. This will contribute to a better understanding of morphological diversity within the group, allow functional interpretation, and permit some evaluation of the muscle traits associated with an unfenestrated and almost non-emarginated skull.

### Phylogenetic Background

The origin of turtles (total group Testudinata) and their phylogenetic relationship to other amniotes remains contentious (e.g. [42], [58]–[60]). Although the detailed arrangement of individual bones differs, the enclosed temporal region of sea turtles is superficially comparable to that of many extinct non-amniotes and early reptiles. Consequently, turtles were commonly considered to represent a surviving lineage of early anapsids such as diadectids, captorhinids, *Eunotosaurus*, pareiasaurs, or procolophonids (e.g. [61]–[69]). Although this interpretation is often labelled the “traditional” hypothesis (e.g. [59], [70]–[74]), there are several examples of pre-cladistic and premolecular studies that instead argued in favour of a closer affinity between turtles and diapsids based on morphological data (e.g. [45]–[47], [75]–[79]).

Early cladistic studies placed turtles within anapsids close to or within captorhinids, pareiasaurs, or procolophonids (e.g. [80]–[82]). However, comparisons were largely restricted to Palaeozoic taxa. Subsequent, more inclusive analyses recovered turtles within Diapsida, as a sister taxon of lepidosaurs (lizards, snakes, and tuatara) (e.g. [83]–[87]). Molecular analyses have also favoured an origin within Diapsida but repeatedly as the sister taxon of archosaurs (crocodiles and birds) (e.g. [71], [72], [74], [88], [89]). Morphological evidence congruent with this hypothesis does exist but is rather meagre (e.g. [79], [90]–[92]). Combined morphological-molecular studies to date, appear to mainly support an anapsid origin [70], [73]. Evidence from embryological data suggests an origin from outside Sauria (Lepidosauria+Archosauria) [42]. Other recent morphological studies [58], [93], which include a rexamination of the Palaeozoic reptile *Eunotosaurus* [64], [94], found renewed support for the anapsid origin of turtles. However, the most recent molecular analyses support an affinity with either lepidosaurs (mRNAs: [59]) or again archosaurs (nDNA: [60]).

As stated above, the precise phylogenetic position of Chelonioidea within crown turtles (Testudines) remains uncertain [39]–[44]. A recent analysis by Joyce [40] based on morphological data found Chelonioidea as the sister taxon to all other cryptodires (Fig. 1A), whereas Shaffer [41] using molecular evidence placed Chelonioidea nested within cryptodires as the sister taxon to a clade that includes Chelydridae and Kinosternidae (Fig. 1B).

Phylogenetic relationships within Chelonioidea also remain unresolved [21], [40], [43], [44], [95]–[98]. For example, the results of recent phylogenetic analyses of Chelonioidea disagree as to whether or not *Ch*. *mydas* is the sister taxon of *E*. *imbricata* (Fig. 1 C, D) [23], [40], [43]. However, there is consensus that *D*. *coriacea* is the sister taxon to all remaining living sea turtles (Cheloniidae), as well as support for a grouping of *C*. *caretta* and *L*. *kempii* + *L*. *olivacea* [23], [41], [43], [98], [99] (Fig. 1 C, D), a clade referred to as Carettini [2], [3], [23]. Mitochondrial sequences suggest that *C*. *caretta* and *L*. *kempii* diverged from one another during the Miocene (10–20 Ma, [98], [99]), a conclusion that agrees with previous inferences based on fossil data [3]. These two turtles are similar in their external morphology but the former tends to be much larger, possesses four inframarginal scales rather than three, lacks pores on these scales, and has maxillae that meet in the midline of the palate [7], [29], [40], [100], [101].

### Ecological Background

The loggerhead turtle, *C*. *caretta*, is widely distributed and nests on a number of subtropical beaches along the coasts of the Mediterranean, eastern USA, Cape Verde Islands, Brazil, Africa, Oman, Japan, Australia, and various Caribbean islands [1], [12], [102]–[105]. It is a relatively large turtle with adults attaining carapace lengths of 85–124 cm and weights of 80–200 kg, although individuals described in historical reports greatly exceeded this [1], [12]. Loggerheads mainly feed on sea pens, crabs, and molluscs, but there are proportional differences in diet according to ontogenetic age and the time of the year [1], [25], [106]–[110]. This is partly because juveniles tend to live in the open ocean whereas adults spend more time closer inshore where available food sources are different [25], [104]. Bite force measurements are currently unavailable, but large adults clearly have powerful jaws [1], [12] as, for example, Bustard [1] reports an individual biting through a clam shell 8 mm thick. *Caretta caretta* is important to marine-terrestrial food webs [1], [12], [102], [111]–[114] as well as the tourist industry of both Greece and Florida [115]. Furthermore, it is of conservation concern [1], [102], [115].

The Kemp’s ridley, *L*. *kempii*, is less well known and is considered to be the rarest and most endangered sea turtle [1], [6], [12], [116], [117]. It nests on Atlantic beaches in Mexico and Texas but can be found far eastward into the Atlantic [12]. It is generally smaller than *C*. *caretta*, with adults having carapace lengths of 61–76 cm and weights of 36–46 kg [12]. As with *C*. *caretta*, juveniles of *L*. *kempii* tend to live in the open ocean whereas adults spend more time closer inshore [25], [118]; they are thought to feed mainly on crustaceans (such as portunid crabs) and molluscs [12], [25], [100], [107], [119].

### Institutional Abbreviations

LACM, Natural History Museum of Los Angeles County; LDUCZ, Grant Museum of Zoology, University College London, UK; UMZC, University Museum of Zoology, Cambridge, UK.

## Results

### General Head Anatomy and Osteology of the Cranium

The skull of *Caretta caretta* has been described by Carr [120], Gaffney [29], Kamezaki [101], and Arenciba *et al*. [57], whereas that of *Lepidochelys kempii* has been described by Hay [26], Carr [120], and Matzke [22]. A more general description of sea turtle skull morphology was provided by Wyneken [8], [100].

**Figure 2:**
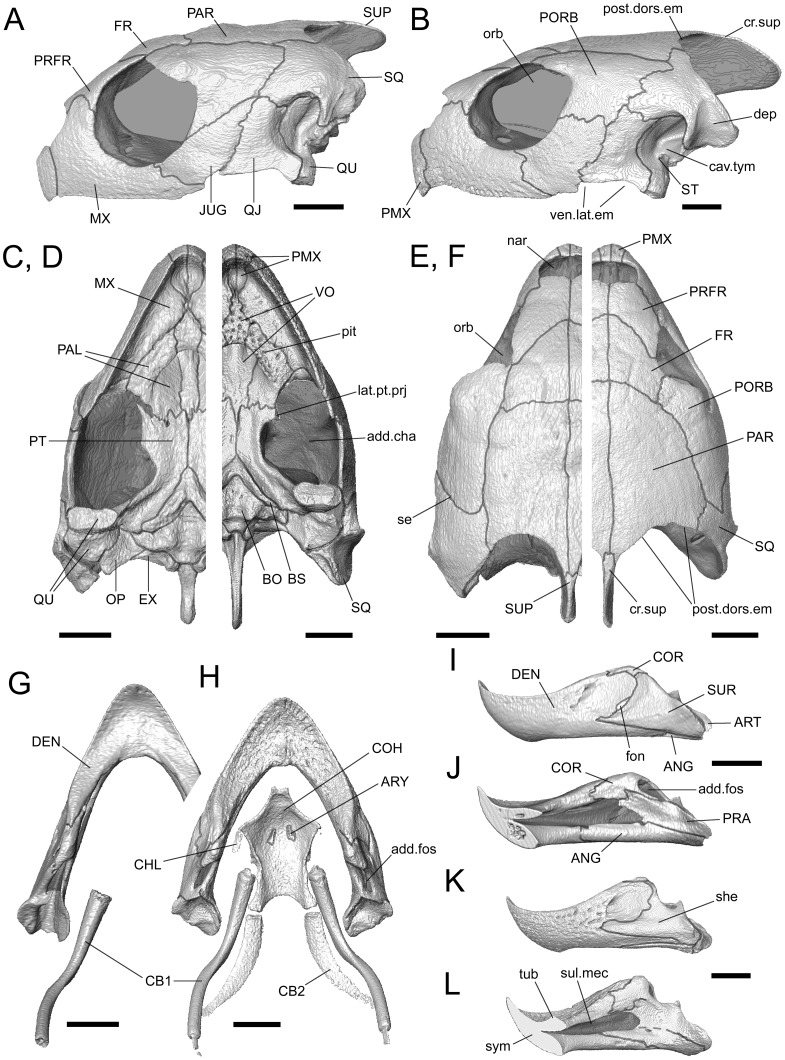
The skulls of two sea turtles. (**A**, **C**, **E**, **G**, **I**, and **J**) *Caretta caretta* (XT757/07) and (**B**, **D**, **F**, **H**, **K**, and **L**) *Lepidochelys kempii* (M009/08). The cranium in (**A** and **B**) lateral, (**C** and **D**) palatal, and (**E** and **F**) dorsal views. The lower jaws and hyoid skeleton in (**G** and **H**) in dorsal view. The left lower jaw in (**I** and **K**) lateral and (**J** and **L**) medial view. add. fos, adductor fossa, add.cha, adductor chamber, ANG, angular; ART, articular; ARY, arytaen; BO, basioccipital; BS, basisphenoid or sphenoid; cav.tym, cavum tympanicum; CB1, cornu branchial-I; CB2, cornu branchial-II; CHL, cornu hyale; COH, corpus hyoidei; COR, coronoid bone; cr.sup, crista surpaoccipitale; DEN, dentary; dep, depression; EX, exoccipital; Fon, fontanelle; FR, frontal; JUG, jugal; MX, maxilla; OP, opisthotic; orb, orbit; PAR, parietal; pit, pits; PMX, premaxilla; PORB, postorbital; post.dors.em, posterodorsal emargination; PRA, prearticular; PRFR, prefrontal; PT, pterygoid; PAL, palatine; add.cha, adductor chamber; nar, narial opening; QJ, quadratojugal; QU, quadrate; se, suture seam; she, shelf; SQ, squamosal; ST, stapes (columella); sul.mec, sulcus Meckeli; SUP, supraoccipital; SUR, surangular; sym, symphysis; tub, tubercle; vent.lat.em, ventrolateral emargination; VO, vomer. Scale bars = 10 mm.

As in *Chelonia mydas* [121], both taxa show allometric variation in skull shape (particularly *C*. *caretta*, pers. obs. M.E.H.J.) but this has yet to be fully surveyed. The variation makes it difficult to identify diagnostic skull shape differences between the two taxa that are size independent, especially because adult *C*. *caretta* are much larger than adult *L*. *kempii*, many published images lack scale bars or measurements (e.g. [8], [101]), and geographic variation is not fully understood in these taxa. Nevertheless, the skull of an immature *C*. *caretta* does not entirely resemble a similar sized skull of *L*. *kempii* (Fig. 2A, B).

**Figure 3:**
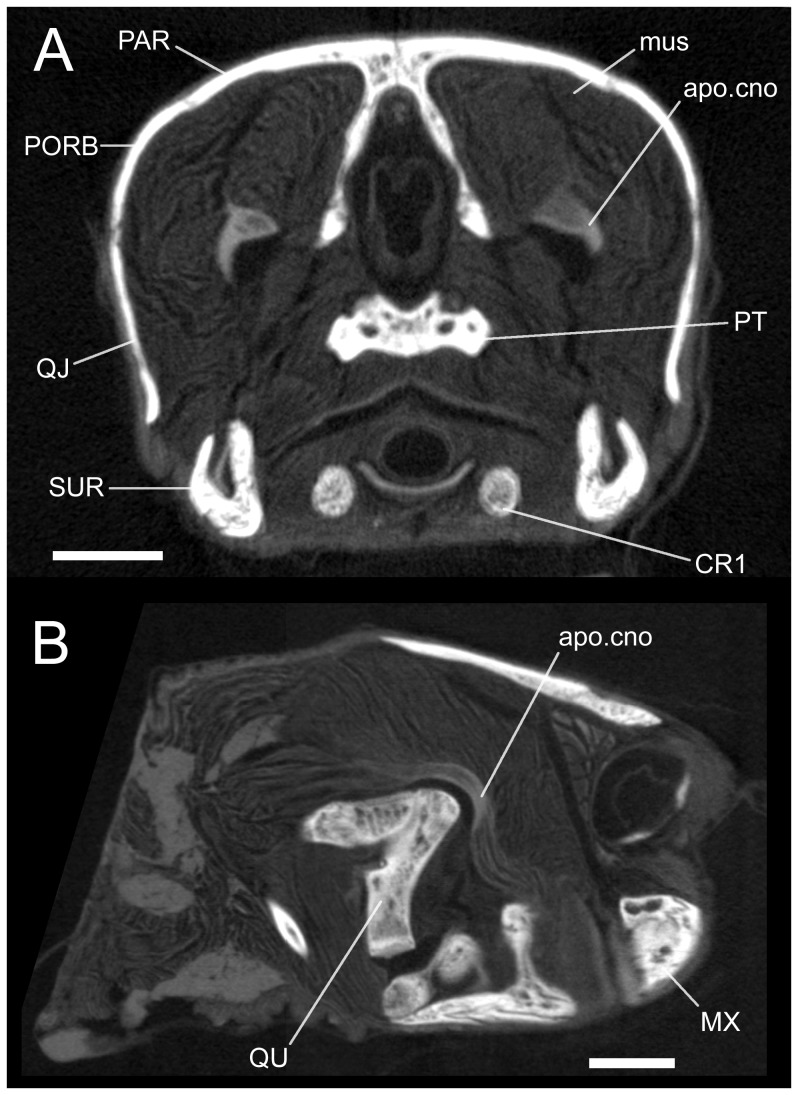
Micro-CT slices of the sea turtle *Lepiodochelys kempii* (M009/08) showing muscle structures. (**A**) coronal slice 520 of 980 and (**B**) parasagittal slice 193 of 675. apo.cno, coronar aponeurosis; CB1, cornu branchial-I; CB2, cornu branchial-II; MX, maxilla; mus, muscular structures; PAR, parietal; PORB, postorbital; PT, pterygoid; QJ, quadratojugal; QU, quadrate; SUR, surangular. Scale bars = 10 mm.

**Figure 4:**
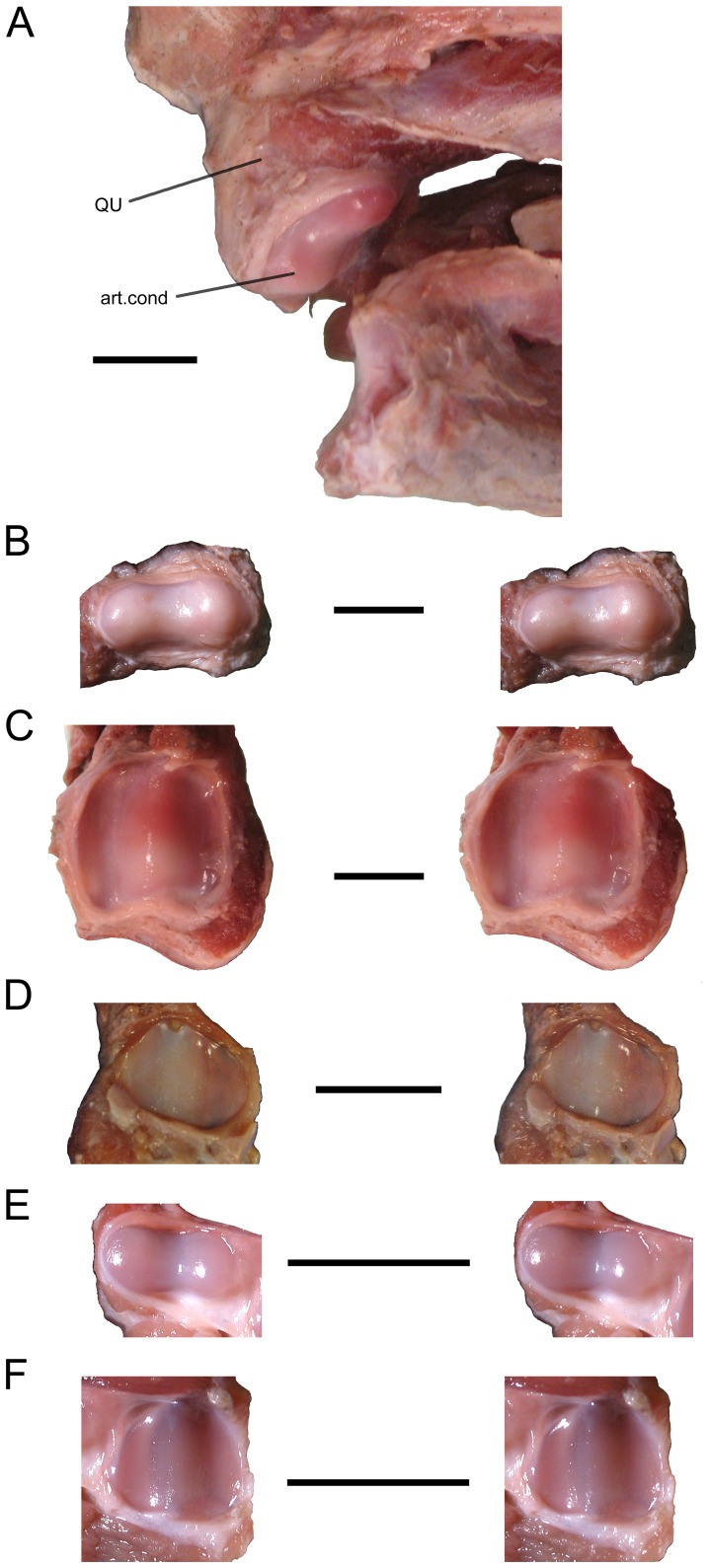
The jaw joints of two sea turtles. (**A–D**) *Caretta caretta* and (**E–F**) *Lepidochelys kempii*. The left quadrate condyle of specimen XT161/08 in (**A**) ventromedial view. Stereopairs of the (**B**) left quadrate condyle and (**C**) left articular of specimen XT161/08; (**D**) right articular surface of specimen XT144/08; and the (**E**) left quadrate condyle, and (**F**) left articular of specimen XT043/08. art.cond, articular condyle; QU, quadrate. Scale bar = 10 mm.

The skulls of both *C*. *caretta* and *L*. *kempii* are dome-shaped (Fig. 3), taper towards the rostrum and, compared to other Testudines, possess relatively small ventrolateral and posterodorsal emarginations (Figs. 2A, B; [8], [26], [29], [101], [120], [122]). In *C*. *caretta*, the temporal region (posterior portion of the cranium) is relatively large compared to the snout region (Figs. 2A, C, E) and the difference becomes greater with size (specimens LDUCZ×11; UMZC R 4606; Video S1, S2; Table 1, [8], [29]). In both taxa, the rostral tip of the skull is formed by a pair of premaxillae that lack any kind of dorsomedial projection so that the nares are confluent (Figs. 2E, F). The maxilla has a well-defined facial process and a deep suborbital margin (Figs. 2A, B). As in all extant turtles, the lacrimal is absent [29]. The jugal lies at the posteroventral corner of the orbit and connects to the quadratojugal posteriorly and the postorbital dorsally (Figs. 2A, B). In both taxa, the posteroventral corner of the jugal occasionally bears a caudal spur (e.g. [8]: fig. 40), the size of which can vary between left and right sides (e.g. specimen M009/08, Table 2). In small specimens of *C*. *caretta* contact between the postorbital and quadratojugal in lateral view is almost excluded by the jugal and squamosal (e.g. XT757/07; LDUCZ×1183; Figs. 2A). This is probably due to ontogenetic variation because contact is obvious in larger skulls (e.g. LDUCZ×11; UMZC R 4606; UMZC R 4606; [120]). The quadrate is located posterior to the quadratojugal, with its concave lateral surface forming the majority of the cavum tympanicum (Figs. 2A, B; [123]), which is associated with the auditory apparatus. Posteroventrally, the quadrate is rugose, particularly in large skulls (e.g. UMZC R 4606). The ventral end of the quadrate bears a relatively shallow condyle formed from cartilage. The two lobes are almost symetrical but the slightly smaller lateral lobe projects more ventrally (Figs. 4A, B; XT161/08).

**Table 1:** Skeletal material examined. Skull length is measured along the midline from the tips of the premaxillae to a point level with the back of the quadrates. Units in mm.

Specimen number	Taxon	Skull length	Material
UMZC R 4606	*Caretta caretta*	245	cranium and lower jaw
LDUCZ×11	*Caretta caretta*	186	cranium and lower jaw
UMZC R 4609	*Caretta caretta*	128	cranium, sawn parasagitally
LDUCZ×1183 (was UCL Z)	*Caretta caretta*	107	cranium and lower jaw
UMZC R 4611	*Lepidochelys olivacea*	140	cranium
LDUCZ×34	*Lepidochelys* sp.	123	cranium and lower jaw
LDUCZ×1184 (was LDUCZ×132)	*Lepidochelys* sp.	116	cranium and lower jaw
LACM 164121	*Lepidochelys olivacea*	48	cranium, lower jaw, and rhamphothecae

**Table 2:** Specimens dissected or CT scanned. Measurements taken correspond to those listed in Wyneken [8] as far as possible.

Ref no.	Specimen no.	Taxon	Sex	Skull length	Neck Circumferance	Stright overall length (SOL)	Linear shell length (SCL)	Straight carapace width (SCW)	Weight	Origin
T2007/06	XT757/07	*C*. *caretta*	?	50	165	270	190	190	1.426	Tywyn, Gwynedd, Wales
T2008/10	XT144/08	*C*. *caretta*	M	57	155	330	250	220	1.994	Cae Du, Gwynedd, Wales
T2008/13	XT161/08	*C*. *caretta*	F	110	260	590	440	400	11.4	Black Rock Sands, Gwynedd, Wales
T2008/2	XT043/08	*L*. *kempii*	M	48	150	255	195	180	1.185	Porth Ceiriad, Gwynedd, Wales
T2008/017	M009/08	*L*. *kempii*	F?	58	160	320	240	210	1.8	Berbencula, Western Isles, Scotland

All measurements except skull length were taken by the Cetacean Strandings Investigation Programme (CSIP), UK. The prefixes XT and M denote whether the postmortem was carried out by the Institute of Zoology, London, UK, or the Animal Health and Veterinary Laboratories Agency, Truro, UK, respectively. Skull length is measured along the midline from the rostrum parallel to a point level with the back of the quadrate condyle. Units in mm or kg.

The squamosal is situated on top of the quadrate and quadratojugal and bears a lateral depression on its posterolateral corner (Figs. 2A, B: LDUCZ×11); in similar-sized skulls of *C*. *caretta* and *L*. *kempii*, it is better defined in the latter species (Fig. 2B). The skull roof comprises paired parietals, frontals, and prefrontals of which the parietals form the largest component (Figs. 2E, F, 3; [8], [26], [59], [29]). In the smallest specimen of *C*. *caretta* the external junction between the frontals, prefrontal, and postfrontal is very close to the orbital margin (XT757/07; Fig. 2E). In larger specimens the junction occurs more medially so that the frontals are fully excluded from the border of the orbit by the prefrontal and postfrontal (e.g. LDUCZ×1183; LDUCZ×11; UMZC R 4606; UMZC R 4609). This character does not generally occur in *L*. *kempii*, *L*. *olivacea*, *Ch*. *mydas,* or *Eretmochelys imbricata* (Fig. 2F; [8], [22], [26], [120]) but it is present in one specimen thought to represent a species of *Lepidochelys* (LDUCZ×34). Large specimens of *C*. *caretta* possess a prominent spine extending from the posterior edge of the squamosal (e.g. LDUCZ×11; UMZC R 4606). In *C*. *caretta* and *L*. *kempii*, the squamosals meet the parietals and, medial to this, the supraoccipital projects posteriorly to form the crista supraoccipitale (Figs. 2A, B, E, F) but it is relatively larger in *L*. *kempii* (Figs. 2B, F). Within the adductor chamber the medial process of the quadrate and lateral process of the prootic meet to form the processus trochlearis otici.

The palatal arrangement is similar in both species, but in *C*. *caretta* the maxillae meet along the ventral midline, whereas in *L*. *kempii* and other chelonioids the vomers and premaxillae separate them (Figs. 2C, D; UMZC R 4606; [6], [8], [22], [26], [29], [100], [120], [124], [125]. In both taxa, the palate is reinforced by a central pillar between the vomer and nasal bones and by the vomer-palatine secondary palate above and below the nasal passage [19]. Also, numerous pits can be found on the ventral surface of the palatal bones in larger specimens of both taxa (Fig. 2C; UMZC R 4606). In ventral view the lateral margin of the cranium is curved in *L*. *kempii* rather than sigmoid as it is in *C*. *caretta* (Figs. 2C, D). This is linked to a relatively broader palate and relatively smaller adductor chamber in the former species (Fig. 2C, D; e.g. LDUCZ×11; UMZC R 4606; [8], [22], [26], [101], [120]). As in other cryptodires, a processus trochlearis otici within the chamber is formed by a medial process of the quadrate coonected with the lateral process of the prootic [29]. The lateral pterygoid projection is slightly more pronounced in *L*. *kempii* but it is still small compared to other turtles [22], [29].

The lower jaws are heavily built in both taxa and are fused at the symphysis (Figs. 2G, H; [19], [29]). The dentary projects posteriorly on the labial surface of the jaw alongside the angular, whereas the coronoid and surangular make up the dorsolateral surface (Figs. 2I-L). The lateral surface of the surangular is embayed, particularly in *L*. *kempii*, where a clear shelf occurs ventrally (Fig. 2K). The posterolingual surface is mainly composed of the coronoid and angular bones (Figs. 2J, L). The dorsal surface of the dentary is broad and slightly convex but the obvious ridges found in *Ch*. *mydas* are absent (e.g. UMZC R 4395; [8], [23]). In *L*. *kempii*, the midline of the dentary does bear a dorsal tubercle near its posterior edge which relates to a dorsal spine of the lower rhamphotheca (beak) (M009/08; Figs. 2L). This feature is also present in *L*. *olivacea* (LACM 164121). However, this feature may not be present in adults [19]. In both taxa, pits occur on the dentary dorsally and labially but the extent of these may be size or age related (e.g. Figs. 2H, K). The articular surface for the quadrate is almost flat in both taxa with only a shallow ridge aligned with the long axis of the lower jaw (Figs. 4C, D, F). Although the shape of the outer edges seem to vary with ontogeny (Figs. 4C, D), the surface is approximately twice as long as the corresponding surface on the quadrate condyle. The ligaments around the joint capsule are strong, particularly in the anteromedial, posteromedial, and posterolateral corners. The retroarticular process is very short (Figs. 2G–L).

The heads of both *C*. *caretta* and *L*. *kempii* are covered by scales composed of thickened epidermis and keratin [100], [101]. Moreover, the upper jaw + palate and the lower jaw are covered by keratinous rhamphothecae which have narrow cutting edges and wider surfaces for crushing [8], [100]. In both taxa, the shape of the upper rhamphotheca is related to the shape of underlying bones. Thus, in lateral view the ventral margin of the rhamphotheca is more sigmoid in *L*. *kempii* than it is in *C*. *caretta*.

The hyoid apparatus supports the tongue, pharynx, and floor of the mouth [126], [127]. Its skeletal component comprises a somewhat pentagonal corpus ossis hyoidei located in the throat between the two lower jaws (Fig. 2H; [8]. Along the posterolateral margins are three tubercles (or horns) and, anterior to posterior, these attach to the cornu hyale, cornu branchial-I, and cornu branchial-II (Figs. 2H, 3A). The latter are particularly well-developed and extend posterodorsally around the throat up to a point level with the posterior tip of the quadrate.

### Osteology of the Neck

Within living Testudines, two distinct mechanisms retract and move the neck/head region: the cryptodire condition, where the neck is moved and folded in a vertical plane, and the pleurodire condition, where the neck is moved and folded in a horizontal plane [128]–[130]. Sea turtles are cryptodires, but they have lost the ability to retract their necks [101], [131]. Previous descriptions of cryptodire neck vertebrae include those by Williams [132], Hoffstetter and Gasc [131], Gaffney [133], Meylan and Gaffney [134], and Herrel *et al*. [130]. Testudines have 18 presacral vertebrae of which eight are cervicals [128], [131]. As in most cryptodire turtles, the atlas and axis of sea turtles are not fused and are connected by a ball and socket joint [128]. Vertebra 2 and 3 are opisthocoelous and 4 is biconvex, whereas 5 to 8 are procoelous [131], [134]. The neural spines and transverse processes are relatively short but conspicuous keels project from the ventral surfaces of the centra (e.g. M009/08) [131], [132].

**Figure 5:**
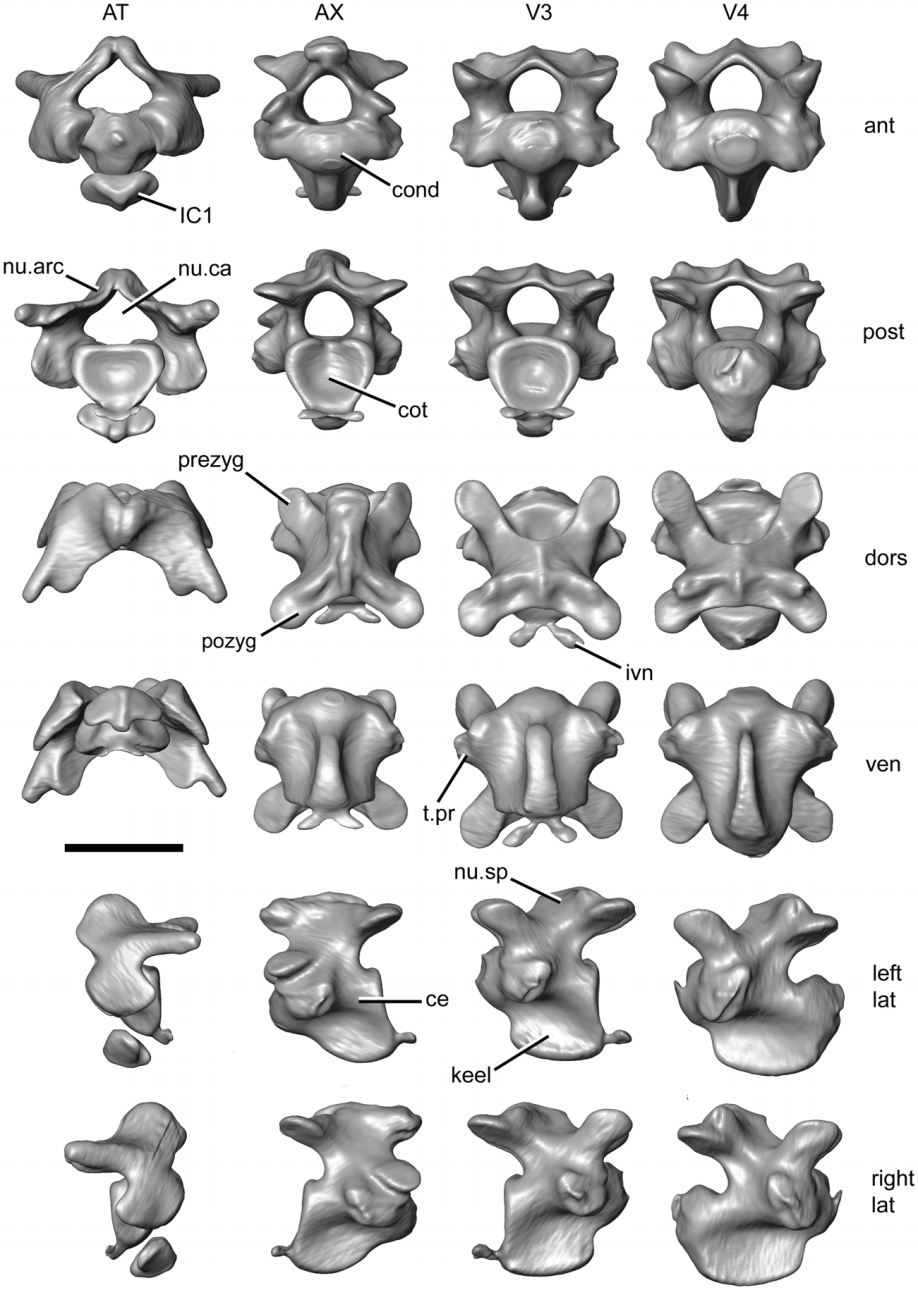
The first four neck vertebrae of the sea turtle *Lepidochelys kempii* segmented from the micro-CT scan of specimen M009/08. ant, anterior; AT, atlas; AX, axis; ce, centrum; cond, condyle; cot, cotyle; dors, dorsal view; ivn, intervertebral nodule; keel, keel; lat, lateral view; nu.arc, neural arch; nu.ca, neural canal; nu.sp, neural spine; post, posterior view; pozyg, postzygapophysis; prezyg, prezygapophysis; t.pr, transverse provess; V3, third vertebra; V4, fourth vertebra; ven, ventral view. Scale bar = 10 mm.

The atlas is notably different from other neck vertebrae. That of *L*. *kempii* (M009/08) has a relatively wide neural arch from which two processes extend posterolaterally over the prezygapophyses of the axis. The atlas centrum and neural arch are anteroposteriorly short (M009/08; Fig. 5). The anterior cotyle appears almost hexagonal in its anterior aspect, whereas the posterior condyle is dorsoventrally compressed and about one third wider than tall. The atlas neural canal is triangular rather than ovoid, as it is in vertebrae 2–5 (Fig. 5). The associated first intercentrum is well-developed and possesses an obvious ventral keel. The axis, third and fourth vertebrae are similar in possessing pre- and postzygapophyses orientated almost parallel to the horizontal plane (M009/08). The third and fourth vertebrae are larger than the axis and have better developed ventral keels anteriorly (Fig. 5). The axis neural spine is not particularly tall but it projects anteriorly and is of equivalent build to the axis ventral keel (Fig. 5). The fourth vertebra is bicondylar (M009/08). The transverse processes are short and oval or sigmoid in cross-section. On the atlas, axis, and third vertebra, small paired processes can be seen to extend posterolaterally from the midline of the posteroventral edge of the centra (Fig. 5). These have previously been termed “intervertebral nodules” [131] and interpreted as the capitular part of vestigal ribs [135]. However, we suggest that their location is more congruent with them representing vestigal intercentra. The neck of *C*. *caretta* is very similar to that of *L*. *kempii*, but the neural spine of the axis is relatively more pronounced (XT757/07).

### Cranium-Associated Musculature and other Soft Tissue of the Head

Previous descriptions of the feeding related musculature of sea turtles include those of *C*. *caretta* [136], [137], *Ch*. *mydas* [138], [139], *Dermochelys coriacea* [140]–[142], and *E*. *imbricata* [76]. However, our understanding of turtle muscle homologies and nomenclature has progressed since this work was done [143]. Moreover, no jaw muscle descriptions of *Lepidochelys kempii* or *Natator depressus* are known to the authors, and only a few aspects of this anatomy in *L*. *olivacea* have been reported ([144]: 12). For a recent review of previous work, terminology, and muscle synonymies, see Werneburg [143].

Here we follow the system proposed by Werneburg [143] (Table 3), where all cranium-associated muscles of turtles are grouped based on a variety of homology criteria and are given a numerical label (between No. 1 and No. 88. As in Werneburg [143], for anatomical consistency, a ‘muscular unit’ ( =  a muscle *sensu stricto*, s.s., or a muscular portion s.s.) is defined according to possession of its own origin, course, and insertion site. In contrast to muscles s.s., however, muscular portions s.s. show some intercrossing fibres along their course. Muscular units may be partly subdivided into muscle heads. Muscle heads have their own origination or insertion site, but in their course they fuse with other muscle heads to form the discrete belly of a single muscular unit [143]. We do not describe the muscular units related to the nose or eyes (No. 1 to 16, 34–38), blood vessels (No. 48), larynx (No. 49–51), or anterior parts of the tongue (No. 63–74). The following muscular units are not present in either *C*. *caretta* or *L*. *kempii*: No. 18, 20, 22 (but see description of No. 21c), 24, 27 (but see description of No. 26), 30, 32–33, 39, 40, 44, 54, 56, 59, 61–62, 76, 79, and 84–85.

**Table 3:** Muscular unit used in this paper (see also [44], [143], [152]).

Number	Muscular unit
17	m. adductor mandibulae externus Pars medialis
19	m. adductor mandibulae externus Pars profundus
21	m. adductor mandibulae externus Pars superficialis
23	m. adductor mandibulae internus Pars pseudotemporalis principalis
24	m. adductor mandibulae internus Pars pseudotemporalis superficialis
25	m. adductor mandibulae internus Pars intramandibularis
27	m. adductor mandibulae internus Pars pterygoideus posterior
28	m. adductor mandibulae internus Pars pterygoideus ventralis
29	m. adductor mandibulae posterior Pars principalis
31	m. intermandibularis
41	m. constrictor colli Pars spinalis
42	m. constrictor colli Pars intermandibularis
43	m. constrictor colli Pars oralis
45	m. depressor mandibulae
46	m. dilatator tubae
47	m. branchiomandibularis visceralis
52	m. plastrocapitis
53	m. squamosobranchiale
55	m. branchiohyoideus
57	m. collosquamosus
58+60	m. coracohyoideus
75	m. atlantoepistropheooccipitis
77	m. atlantooccipitis
78	m. atlantoopisthoticus
80	m. collooccipitalis
81	m. testocapitis
82	m. testooccipitis
83	m. transversalis cervicis
86+87	m. longus colli Partes capitis-I et Pars capitis-II/III
88	m. retrahens capiti collique Pars carapacobasioccipitalis

In the following account, the anatomy of *C*. *caretta* is usually described first followed by the differences found in *L*. *kempii* where present.

### Ligamentum Quadratomaxillare

**Figure 6:**
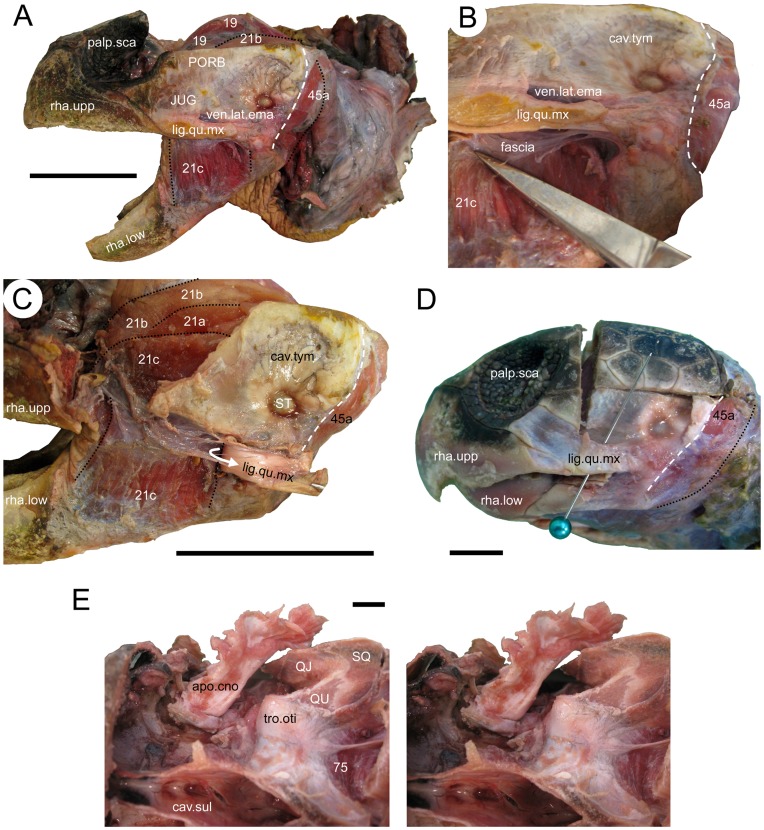
The ligamentum quadratomaxillare and coronar aponeurosis in two sea turtles. The quadratomaxillar ligament in (**A**) *Caretta caretta* (XT161/08) in lateral view, (**B**) ventrolateral view to show its connection to the lower temporal fascia, and (**C**) after being cut and peeled back to show its medial surface. (**D**) the ligamentum quadratomaxillare in *Lepidochelys kempii* (XT043/08). (**E**) a stereo pair of the coronar aponeurosis in *Caretta caretta* (XT161/08). 19, the m. adductor mandibulae externus Pars profundus; 21a, m. adductor mandibulae externus Pars superficialis medial head; 21b, m. adductor mandibulae externus Pars superficialis posterior head; 21c, m. adductor mandibulae externus Pars superficialis lateral head; 45a, m. depressor mandibulae lateral part; 75, m. atlantoepistropheooccipitis; apo.cno, coronar aponeurosis (external tendon); cav.sul, cavum sulcus; cav.tym, cavum tympani; fascia, fascia; JUG, jugal; lig.qu.mx, ligamentum quadratomaxillare; palp.sca, palpebral scales; PORB, postorbital; QJ, quadratojugal; QU, quadrate; rha.low, lower rhamphotheca; rha.upp, upper rhamphotheca; SQ, squamosal; ST, stapes (columella); tro.oti, processus trochlearis otici; ven.lat.ema, ventrolateral emargination. Scale bars: (A) 50 mm; (C and G) 10 mm.

Although this structure in turtles is refered to as the “ligamentum quadratomaxillare” (e.g. [76], [143]) it can involve contact with the jugal rather than the maxilla. After removal of the temporal scale, in both *C*. *caretta* and *L*. *kempii* this large ligament is visible between the jugal and the jaw joint (quadratojugal/quadrate) below the shallow ventrolateral emargination (Figs. 6A–D). As in other turtles, a fascia is present (Fig. 6B; fascia temporalis anterostegalis of Werneburg [143]) between the ventral margin of the skull and the medial surface of the ligament.

### The Coronar Aponeurosis

The coronar aponeurosis (*sensu* Iordansky [145]  =  Bodenaponeurose *sensu* Lakjer [76]  =  bodenaponeurosis *sensu* Rieppel, [146]  =  external tendon e.g. *sensu* Schumacher [126]; see Werneburg [143] for further synonyms) a large arponeurosis to which some of the external adductor muscles insert. It has previously been described as attaching to the dorsal, medial, and/or lateral face of the coronoid, dentary, and surangular [136], [137], [143], [145]. It attaches directly and broadly to the coronoid in *C*. *caretta* and extends posterodorsally into the adductor chamber passing over the processus trochlearis otici (Figs. 3B, 6E). A cross-section through the left aponeurosis in specimen XT161/08 measured 13 mm wide by 9 mm tall. The aponeurosis in *L*. *kempii* follows a similar path (M009/08). Direct examination and removal of the surrounding muscles reveals the aponeurosis to be broadly trapezoidal in medial view with the anteroventral corner inserting onto the coronoid (Fig. 6E; XT161/08). In lateral view, a thin, strap-like sheet extends laterally from the posterolateral surface and a triangular fold near the base. This fold is less obvious in *L*. *kempii*.

### M. Adductor Mandibulae Externus (No. 17–21)

The m. adductor mandibulae externus (No. 17–21, see Table 3) is innervated by n. trigeminus (cranial nerve V) and is located between the maxillary (V_2_) and mandibular (V_3_) branches of n. trigeminus [76], [126], [143]. At its origin, it includes muscular units referred to as Partes medialis (No. 17), profundus (No. 19) et superficialis (No. 21).

**Figure 7:**
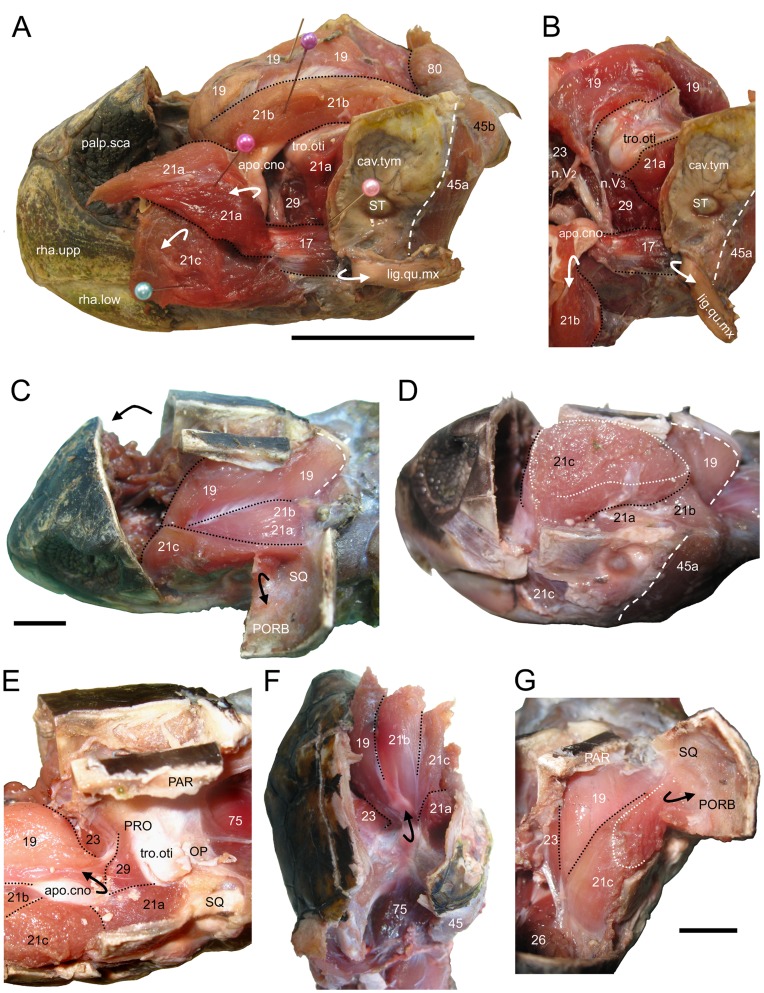
The superficial adductor musculature in two sea turtles. (**A** and **B**) *Caretta caretta* (XT161/08) and (**C** to **G**) *Lepidochelys kempii* (XT043/08). (**A**) lateral view of the head following removal of the skull roof and lateral skull wall. The adductor chamber in (**B**) anterolateral view, (**C**) dorsolateral view, (**D**) posterolateral view, (**E**) dorsolateral view with external adductors folded forwards to show the processus trochlearis otici, (**F**) posterodorsal view, (**G**) anterior view. 17, m. adductor mandibulae externus Pars medialis; 19, m. adductor mandibulae externus Pars profundus; 21a, m. adductor mandibulae externus Pars superficialis medial head; 21b, m. adductor mandibulae externus Pars superficialis posterior head; 21c, m. adductor mandibulae externus Pars superficialis lateral head; 23, the Pars pseudotemporalis principalis; 29, m. adductor mandibulae posterior Pars principalis; 45a, m. depressor mandibulae lateral part; 45b, m. depresor mandibulae, medial part; 75, m. atlantoepistropheooccipitis; apo.cno, coronar aponeurosis (“external tendón”); cav.tym, cavum.tym; clu, columella; lig.qu.mx, ligamentum quadratomaxillare; palp.sca, palpebral scales; n.V2, Ramus maxillaris n. trigemini; n.V3, Ramus mandibularis n. trigemini; OP, opisthotic; PAR, parietal; PORB, postorbital; rha.upp, upper rhamphotheca; rha.low, lower rhamphotheca; SQ, squamosal; tro.oti, processus trochlearis otici. Scale bars: (A) 50 mm; (G) 10 mm.

**Figure 8:**
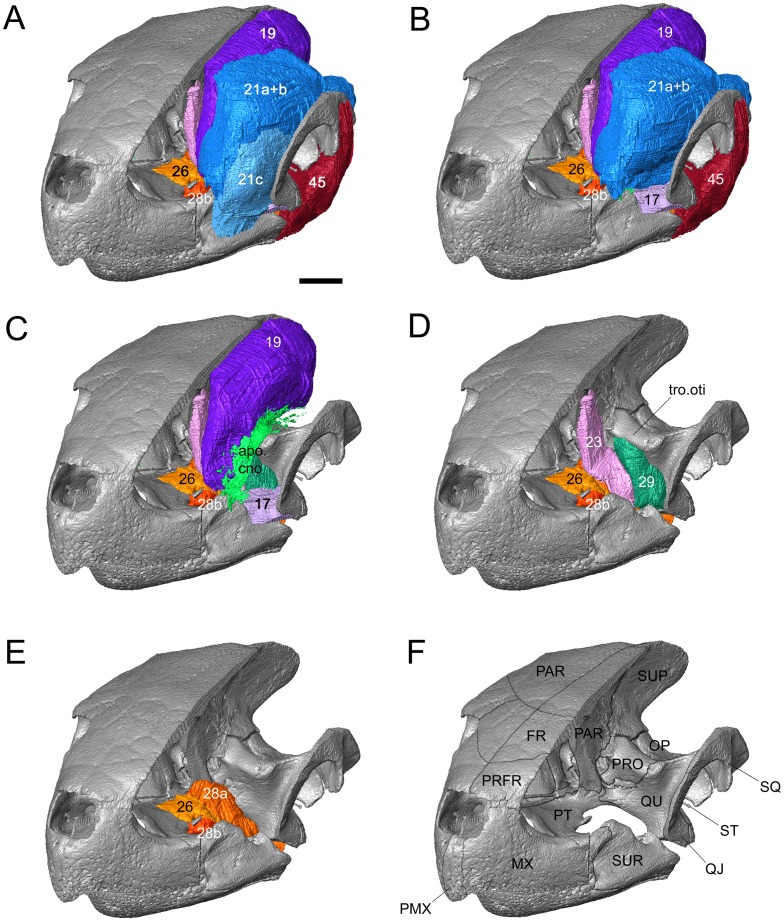
Jaw muscle arrangement in the sea turtle *Lepidochelys kempii* (M009/08) based on reconstructions from micro-CT data. Shown with (**A**) parts of the right side of the skull absent, (**B**) part of the m. adductor mandibulae externus Pars superficialis (No 21c) absent, (**C**) all of the m. adductor mandibulae externus Pars superficialis (No 21abc) and m. depressor mandibulae (No. 45) absent, (**D**) all of the m. adductor mandibulae externus and m. depressor mandibulae (No. 45) absent, (**E**) only the m. adductor mandibulae internus Partes pterygoidei (No. 26, 28) and (**F**) all muscle absent. 17, m. adductor mandibulae externus Pars medialis; 19, m. adductor mandibulae externus Pars profundus; 21a, m. adductor mandibulae externus Pars superficialis, medial head; 21b, m. adductor mandibulae externus Pars superficialis, posterior head; 21c, m. adductor mandibulae externus Pars superficialis, lateral head; 23, m. adductor mandibulae internus Pars pseudotemporalis; 26, m. adductor mandibulae internus Pars pterygoideus dorsalis; 29, m. adductor mandibulae posterior Pars principalis; 45, m. depressor mandibulae**;** apo.cno, coronar aponeurosis (“external tendón”); FR, frontal; MX, maxilla; OP, opisthotic; PAR, parietal; PRFR, prefrontal; PRO, prootic; PT, pterygoid; QJ, quadratojugal; QU, quadrate; SQ, squamosal; ST, stapes; SUP, supraoccipital; SUR, surangular; tro.oti, processus trochlearis otici**.** Scale bar = 10 mm.

The m. adductor mandibulae externus Pars medialis (No. 17), was previously figured by Schumacher ([137]: table 7/2) as the “m. adductor medialis externus portio medialis” (for synonyms see [143]). In accordance with this previous work, our specimens show that this muscular unit is relatively small and belt-shaped (Figs. 7A, B). It originates from the lateral half of the anteroventral face of the quadrate, exactly above the ventrolateral condyle of the quadrate, passes anteriorly, and inserts by a mixture of direct fibres and tendinous structures into the posterodorsal edge of the surangular. This muscular unit is similar in both *C*. *caretta* and *L*. *kempii* (Fig. 8A) but in the latter taxon Pars medialis (No. 17) is not as clearly separated from Pars superficialis (No.21) and some fibres also insert onto the medial face of the surangular.

According to Schumacher [126], the m. adductor mandibulae externus Pars profundus (No. 19) is usually the largest part of the m. adductor mandibulae externus complex (No. 17–19) in turtles. In *C*. *caretta*, Schumacher [137] described the Pars profundus as originating from the crista supraoccipitalis, the lateral face of the supraoccipital, and the lateral face of the processus descendens parietalis, and reaching caudally over the Pars medialis (No. 17) and the caudal end of the supraoccipital. Similarly, Poglayen-Neuwall [136] reported that the Pars profundus originates from the lateral face and the dorsal edge of the parietal and supraoccipital, and the edge of the temporal emargination. The Pars profundus (No. 19) reportedly inserts on the dorsal and ventral faces inside a groove formed by the main body of the coronar aponeurosis and a medial fold [136]. In our specimens, this muscular unit is the largest portion of the m. adductor mandibulae externus complex (No. 17–19) and dominates the medial half of the adductor chamber (Fig. 7). We found the Pars profundus (No. 19) to originate on the lateral face of the crista supraoccipitalis and the posterior part of processus descendens parietalis. The muscle passes anteroventrally and inserts on the dorsal and ventral faces of the coronar aponeurosis involving two heads (19a and 19b) partially divided by a slip of soft tissue (XT161/08). The medial head (No. 19a) originates from the parietal and crista supraoccipitalis and inserts on the ventromedial part of the coronar aponeurosis. The lateral head is smaller (No. 19b). It originates from the back of the adductor chamber (parietal and supraoccipital) and inserts on the dorsomedial surface of the coronar aponeurosis. In *L*. *kempii* (Figs. 7 and 8) the muscular unit is undivided and insertion occurs on the medial surface of coronar aponeurosis.

The m. adductor mandibulae externus Pars superficialis (No. 21) represents the third unit of m. adductor mandibulae externus (No. 17–21). Pars superficialis lies in the lateral area of the postorbital adductor cavity, partially covering the Pars medialis (No. 17) caudally, but previous descriptions of its origin and insertion in *C*. *caretta* conflict.

As described by Schumacher [126], the fibres originate from the parietal in *C*. *caretta* and attach to the dorsal or lateral surface of the coronar aponeurosis. Other descriptions (e.g. Schumacher [137]) indicate the origin as being from the lateral temporal face of the parietal, the caudal end of the postorbital, the medial face of the squamosal, the dorsal face of the quadrate, and the dorsal part of quadratojugal. A small part is also sometimes considered to originate from the dorsal area of the jugal (Schumacher [137]). Alternatively, Poglayen-Neuwall [136] documented that the origin comprised the rostromedial face of the quadrate, the temporal fascia, and the medial face of the quadratojugal and the jugal. In addition, rostral fibres are said to originate from the posteromedial face of the postorbital and rostral part of the squamosal. The insertion has been described as on the lateral edge of the coronar aponeurosis with more superficial fibres attaching directly to the dorsolateral surface of the surangular and the coronoid of the lower jaw (Schumacher [137]). Alternatively, the muscle is reported to insert directly on the dorsolateral surface of the mandible (surangular and coronoid) with some deep fibres inserting on the lateral face of the coronar aponeurosis (Poglayen-Neuwall [136]).

**Figure 9:**
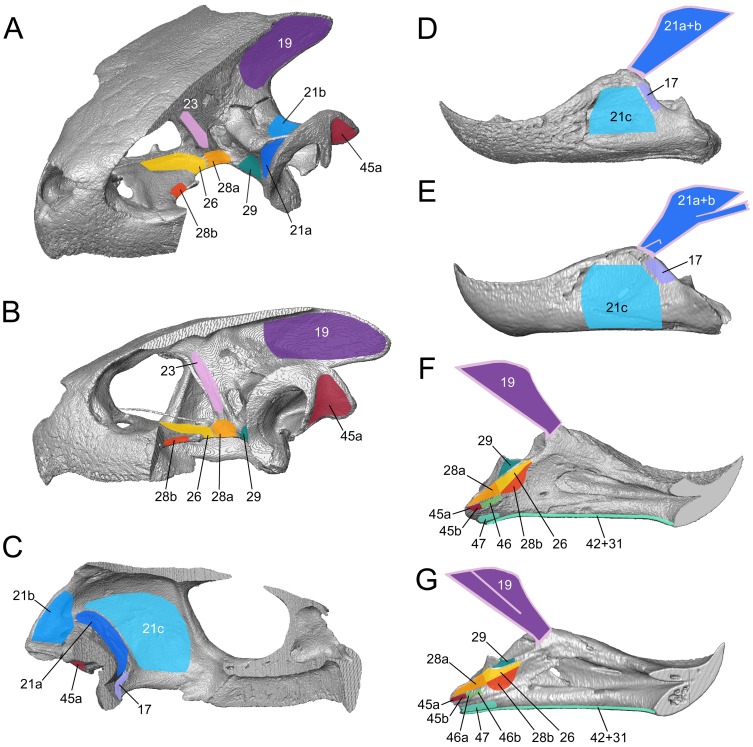
The origin and insertions of jaw muscles in two sea turtles illustrated using computer models based on micro-CT data. (**A–D** and **F**) *L*. *kempii* (M009/08) and (**E** and **G**) *C*. *caretta* (XT757/07). (**A**) dorsolateral and (**B**) lateral view of the cranium with the temporal region removed. (**C**) medial view of the temporal region of the left cranium. (**D**) and (**E**): lateral views of the left lower jaw. (**F**) and (**G**) medial views of the left lower jaw. 17, m. adductor mandibulae externus Pars medialis; 19, m. adductor mandibulae externus Pars profundus; 21a, m. adductor mandibulae externus Pars superficialis medial head; 21b, m. adductor mandibulae externus Pars superficialis posterior head; 21c, m. adductor mandibulae externus Pars superficialis lateral head; 23, m. adductor mandibulae internus Pars pseudotemporalis principalis; 24, the Pars pseudotemporalis superficialis; 26, m. adductor mandibulae internus Pars pterygoideus dorsalis; 28a, m. adductor mandibulae internus Pars pterygoideus large medial head; 28b, m. adductor mandibulae internus Pars pterygoideus small lateral head; 29, m. ductor mandibulae posterior Pars principalis; 45a, m. depressor mandibulae lateral part; 45b, m. depressormandibulae medial part; 46, m. dilator tubae; 47a, m. branchiomandibularis visceralis small posterior head; 47b, m. branchiomandibularis visceralis large medial head; 75, m. atlantoepistropheooccipitis. Scale bars = 10 mm.

Our observations do not precisely match previous descriptions. In *C*. *caretta*, the m. adductor mandibulae externus Pars superficialis (No. 21) was found to originate by three distinct heads within the lateral part of the adductor chamber (Fig. 7A). One head (No. 21a) originates from the anterior face of the quadrate (Fig. 7B). A second head (No. 21b) originates from the posterior edge and the posterodorsal surface of the squamosal (Fig. 7A), with some fibres arising laterally from the posterior face of the opisthotic (XT161/08). A third head (No. 21c) is lateral to the others and narrow in the parasagittal plane (Fig. 7A). The third head originates from the medial surface of four temporal bones: the anterior part of the quadratojugal, the anterior part of the squamosal, the ventral part of the postorbital, and the posterior part of the jugal (XT161/08). The longest fibres originate slightly above the dorsalmost point of the cavum tympanicum (XT144/08). The first (No. 21a) and second (No. 21b) heads converge anteriorly beyond the processus trochlearis otici. Dorsal fibres of No. 21 a+b insert onto the dorsal surface of the coronar aponeurosis as well as dorsal to a lateral fold. Ventral fibres of No. 21 a+b insert into the ventrolateral face of the coronar aponeurosis as well as the lateral fold (7A). Dorsolateral to the processus coronoideus, the anterior half of the third muscle head (No. 21c) fuses medially with the other heads (No. 21a+b). These fibres insert into the coronar aponeurosis at the base of its attachment to the jaw. In *C*. *caretta* the remainder of the third muscle head (No. 21c) inserts into a broad aponeurosis that is closely apposed to the lateral surface of the lower jaw (surangular) but wraps ventrally to insert on the ventrolateral edge of the dentary and angular (Fig. 9E).

The arrangement in *L*. *kempii* (XT043/08) is slightly different. The medial portions (No. 21a, b) originate from the prootic and opisthotic, but not from the parietal, with the second head (No. 21b) having a relatively larger area of origin posteriorly. The lateral fold in the coronar aponeurosis (to which No. 21 a+b insert) is also less obvious (Fig. 7E, F). Moreover, the lateral head (No. 21c) originates slightly higher on the postorbital and additionally from the squamosal (Fig. 7D, G, and 9C). Also the attachment does not reach as far ventrally as it does in *C*. *caretta*, so that its edge partly coincides with the prominent lateral shelf on the surangular (Fig. 8A, B; see also Video S3).

### M. Adductor Mandibulae Internus (No. 23–28)

The m. adductor mandibular internus (No. 23–28) is innervated by n. trigeminus (V) and is located between its ophthalmic (V_1_) and maxillary branches (V_2_). In the species described here, it includes the Pars pseudotemporalis principalis (No. 23), Pars intramandibularis (No. 25), and two Partes pterygoidei (No. 26, 28) [143].

**Figure 10:**
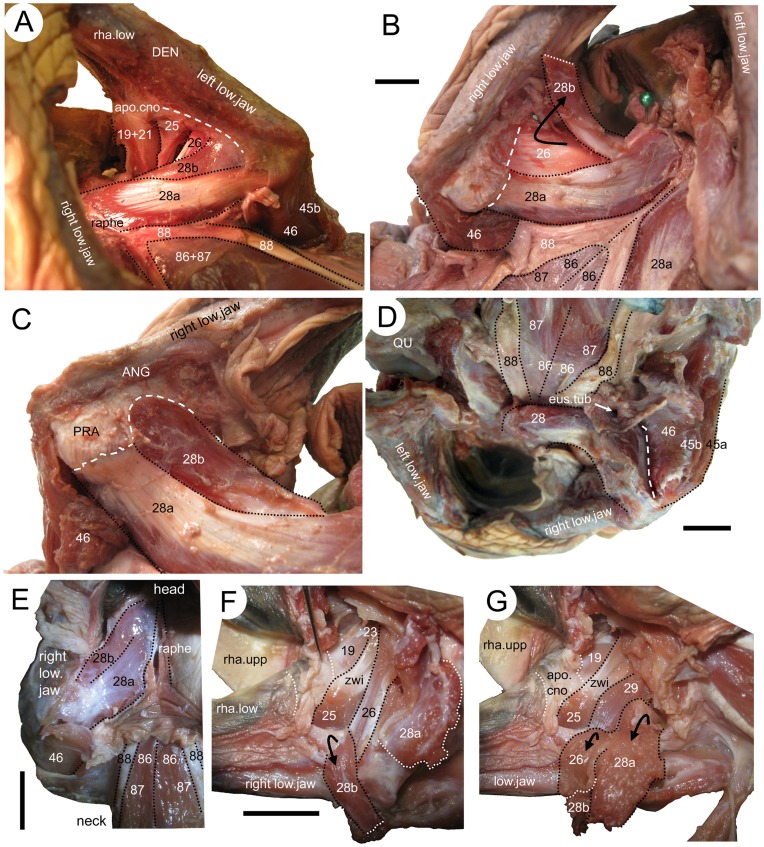
The internal adductor musculature of two sea turtles. (**A**–**D**) ***Caretta caretta*** (XT161/08), and (**E**–**G**) *Lepidochelys kempii* (**XT043/08)**. (**A**) ventrolateral view of the left side adductor mandibulae internus, (**B**) ventral view of the right side internal adductors, (**C**) close up of the right side m. adductor mandibulae internus Pars pterygoideus ventralis, (**D**) posterior view of the internal adductors and hypaxial muscles, (**E**) ventral view of the right side of the palate, (**F**) medial view of the right lower jaw, (**G**) medial view of the right lower jaw after further dissection of the m. adductor mandibulae internus Pars pterygoideus. 19, m. adductor mandibulae externus Pars profundus; 21, m. adductor mandibulae externus Pars superficialis lateral head; 25, m. intramandibularis; 26, m. adductor mandibulae internus Pars pterygoideus dorsalis; 28a, m. adductor mandibulae internus Pars pterygoideus large medial head; 28b, m. adductor mandibulae internus Pars pterygoideus small lateral head; 45a, m. depressor mandibulae lateral part; 45b, m. depressormandibulae medial part; 46, m. dilator tubae; 86, m. longus colli Partes capitis-I et Pars capitis-II; 87, m. longus colli Partes capitis-I et Pars capitis-III; 88, m. retrahens capiti collique Pars carapaco-basiooccipitalis. ANG, angular; apo.cno, coronar aponeurosis; DEN, dentary; low.jaw, lower jaw; PRA, prearticular; QU, quadrate; rha.low, lower rhamphotheca; rha.upp, upper rhamphotheca; raphe, raphe; eus.tub, eustachian tube; zwi, Zwischensehne. Scale bars = 10 mm.

According to Schumacher [126], the “pseudotemporalis muscle” is divided into two units in *C*. *caretta*, the Pars pseudotemporalis principalis (No. 23) and the Pars pseudotemporalis superficialis (No. 24). The former is said to arise from the ventrolateral surface of the parietal and to converge on a tendon which inserts onto the lower jaw posteromedial to the adductor chamber. Posterodorsal to the origin site of Pars superficialis principalis (No. 23), the superficial unit (No. 24) arises from the dorsolateral surface of the processus descendens parietalis. The superficial unit converges on the Zwischensehne (*sensu* [136]), a tendon that connects it with the m. intramandibularis (No. 25). In our specimens of *C*. *caretta*, Pars pseudotemporalis (No. 23) is divided into two portions. Most fibres of this muscular unit originate from the lateral face of the processus descendens parietalis (Figs. 7B, and 8D), but some additional fibres arise from the roof of the adductor chamber (horizontal part of the parietal). All fibres converge into the Zwischensehne (Fig. 10A). In *L*. *kempii*, the anatomy of the Pars pseudotemporalis (No. 23) is similar (Figs. 7F, G, and 8D) but the more dorsally originating muscle fibres are apparently absent and the muscle inserts into a bifurcating Zwischensehne by two separate heads (Fig. 10 F, G).

As described by Poglayen-Neuwall [136] and Schumacher [137], the m. adductor mandibulae internus Pars intramandibularis (No. 25) in *C*. *caretta* originates from the ventral end of the Zwischensehne enters the adductor fossa and inserts into the sulcus Meckeli of the lower jaw. A section through the lower jaw confirms the presence of this muscle in *C*. *caretta* (XT144/08). The same muscle arrangement is also present in *L*. *kempii* (XT043/08) (Fig. 10 F, G).

In *C*. *caretta*, the m. adductor mandibulae internus Pars pterygoideus dorsalis (No. 26) originates from the dorsal surface of the palate (posterior half of the palatine and the dorsal surface of the pterygoid) and from the interorbital septum ventrolaterally, and some fibres may derive from the processus descendens parietalis. The posteriormost fibres may be homologous with the m. adductor mandibulae internus Pars pterygoideus posterior (No. 27). After it passes over the posterior edge of the palatine, the muscular unit inserts on the medial face of the prearticular by a tendinous sheet that is continuous posteriorly with the tendinous sheet of the Pars pterygoideus ventralis (No. 28) (Fig. 10B). Some fibres also insert into the articular and jaw joint capsule. This is broadly similar to previous descriptions ([137]: tabula VII/3a; [126]). In *L*. *kempii*, the absence of the internus Pars pterygoideus posterior (No. 27) is more certain. The Pars pterygoideus dorsalis (No. 26) originates from a smaller area (not including the processus descendens parietalis) (Fig. 8E, and 9A) and inserts via the subarticular aponeurosis into the prearticular on the medial face of the lower jaw (Fig. 8).

The Pars pterygoideus ventralis (No. 28) was described and figured by Schumacher ([137]: table VII/3b) as originating from the ventral and medialmost edges of the pterygoid so that the contralateral portions of No. 28 meet at the palatal midline, separated by a median raphe. In our specimen of *C*. *caretta*, this unit is composed of two muscle heads that are separated superficially but merge at their origin and insertion sites. The larger and more medial head (No. 28a) originates from the posteroventral edge of the palatine and the complete ventral surface of the pterygoid (Fig. 10A, B). As previously described, the contralateral muscle heads (No. 28a) are separated by a midline raphe (Fig. 10A, B). Each head passes ventrolaterally, becoming thinner and inserting into the same tendinous sheet as the Pars pterygoideus dorsalis (No. 26) (Fig. 10B), giving it (No. 28a) a superficially fan-shaped tendinous appearance in ventral view (“Sehnenspiegel” of Schumacher [137]). This tendinous sheet, as well as the deeper muscle fibres, inserts into the medial surface of the prearticular and articular. To reach this position, it passes posteriorly beneath a wide tendon associated with insertion of the m. depressor mandibulae (No. 45). As in the rhynchocephalian lepidosaur *Sphenodon punctatus* [147], some of the posteriormost fibres of the pterygoideus muscle complex insert behind the jaw joint (Fig. 10C; *contra* Schumacher [137]). The smaller muscle head (No. 28b) originates anteriorly from the lateral edge of the pterygoid (Fig. 10A, B). Its fibres pass ventrally, along a fold extending from the tendinous sheet of No. 26 and No. 28a, and have an entirely fleshy insertion into the medial surface of the prearticular ventral to No. 26a and No. 28a (Fig. 9G and 10B).

In *L*. *kempii* (XT043/08), the Sehnenspiegel is not as clear and the fleshy insertion of the smaller head (28b) does not extend as ventrally (Fig. 9F and 10 E).

### M. Adductor Mandibulae Posterior Pars Principalis (No. 29)

This muscular unit is innervated by n. trigeminus (V) and is located posterior to the mandibular branch (V_3_). However, it may not be homologous with the m. adductor mandibulae posterior of some other reptiles because of apparently different developmental origins [143], [146].

The adductor mandibulae posterior Pars principalis (No. 29) has been described as having two heads of origin in *C*. *caretta* [126], [137], with the site of origin of the anterior head reaching dorsally to the posterior border of the processus descendens parietalis [126], [137]. In contrast, this unit (No. 29) originates directly from the anteromedial face of the quadrate in our specimens of *C*. *caretta*, below the processus trochlearis otici and from the complete anterior face of the prootic (Fig. 7B). It extends anteroventrally and inserts on the medial surface of the prearticular – anterior to the joint surface and dorsal to the insertion of the Partes pterygoidei (No. 26, 28) (Fig. 9G). According to Schumacher [126], some fibres would also attach to cartilago Meckeli in *C*. *caretta*, but we could not confirm this. In *L*. *kempii*, the origin is restricted to the anteromedial aspect of the quadrate, with insertion also taking place on the anterior edge of the articular. In medial view it has a tendinous appearance ventrally (Fig. 10G).

### M. Intermandibularis (No. 31)

This muscle is innervated by n. trigeminus (V) and connects the ventral aspects of the lower jaws [143]. Together with m. constrictor colli (No. 41, 42, 43), m. intermandibularis represents a part of the throat musculature. Its anterior fibres reach the ventral edge of the symphysis. Posteriorly it is continuous with the anterior part of the m. constrictor colli Pars intermandibularis (No. 42, see below).

### M. Constrictor Colli (No. 41–43)

**Figure 11:**
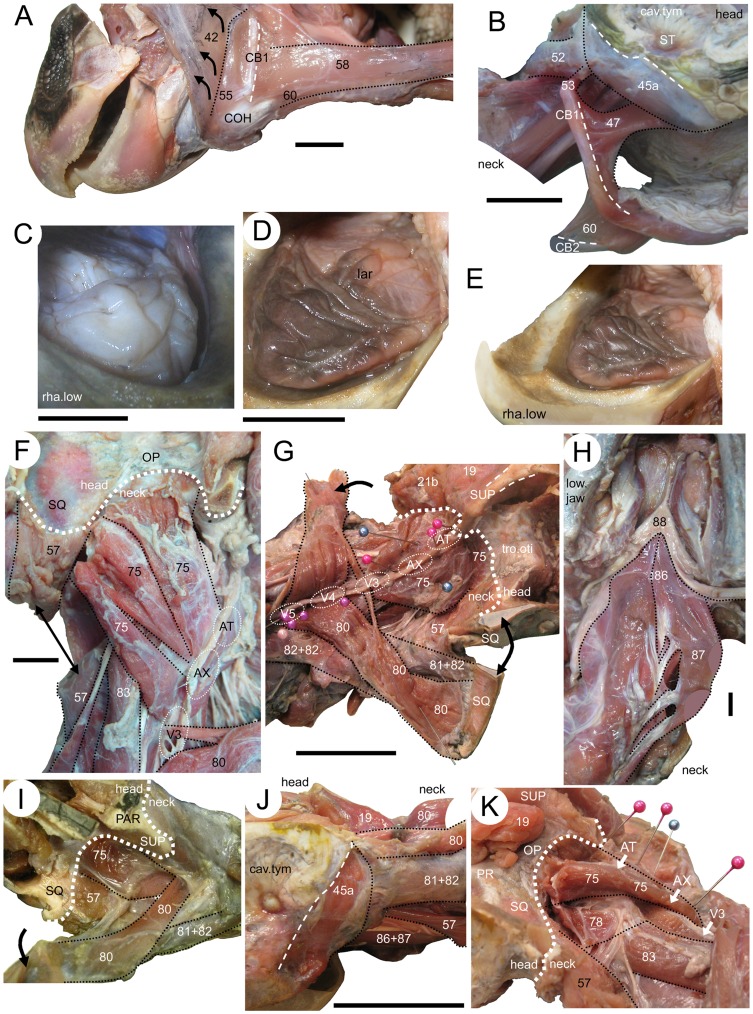
Throat, tongue, and neck muscles of two sea turtles. (**B**, **C**, **F**–**H**, **J**, and **K**) ***Caretta caretta*** and (**A**, **D**, **E**, **I**) *Lepidochelys kempii*. The throat muscles of specimen XT043/08 in (**A**) lateral view of the left side of (**B**) lateral view of the right side. The tongue of (**C**) specimen XT144/08 in dorsolateral view and of (**D**, **E**) specimen XT043/08 in dorsolateral view. The neck muscles of (**F**) specimen XT161/08 in dorsal view of the left side, (**G**) dorsolateral view of the right side, and (**H**) ventral view showing the m. longus colli (No. 86–87). (**I**) dorsolateral view of the right side of specimen XT043/08. Neck muscles of specimen XT161/08 in (**J**) lateral view of the left side, and (**K**) dorsolateral view of the left side. 19, m. adductor mandibulae externus Pars profundus; 21b, m. adductor mandibulae externus Pars superficialis posterior head; 42, intermandibular portion of m. constrictor colli; 45a, m. depressor mandibulae lateral part; 45b, m. depressormandibulae medial part; 46, m. dilator tubae; 47, m. branchiomandibularis visceralis; 52, m. plastro capitis; 53, m. squamosobranchiale; 55, m. branchiohyoideus; 57, m. collosquamosus; 58, m. coracohyoideus main part; 60, m. coracohyoideus small portion; 75, m. atlantoepistropheooccipitis; 80, m. collooccipitalis; 81+82, m. testocapitis et testooccipitis; 83, m. transversalis servicus; 86, m. longus colli Partes capitis-I et Pars capitis-II; 87, m. longus colli Partes capitis-I et Pars capitis-III; 88, m. retrahens capiti collique Pars carapacobasiooccipitalis; AT, atlas (approximate position); AX, axis; cav.tym, cavum tympanicum; CB1, cornu branchial-I; CB2, cornu branchial-II; COH, corpus hyoidei; lar, laryngeal entrance; PAR, parietal; PR, prootic; OP, opisthotic; SQ, squamosal; ST, stapes (columella); SUP, supraoccipital; tro.oti, processus trochlearis otici; cav.tym, cavum typanicum; V3, third vertebra; V4, fourth vertebra; V5, fifth vertebra. Scale bars: (**A**–**D** and **F**) 10 mm; (**G** and **J**) 50 mm.

In contrast to m. intermandibularis (No. 31), the m. constrictor colli complex (No. 41, 42, 43) is innervated by n. facialis (VII). The intermandibular unit of m. constrictor colli (No. 42) lies between the posteroventral edges of the lower jaws and continues anteriorly to m. intermandibularis (No. 31) in both species. A median raphe is visible. The m. constrictor colli Pars spinalis (No. 41) originates from the lateral aspects of the anterior cervical vertebrae and wraps beneath the throat where it inserts into a median raphe. The m. constrictor colli Pars oralis (No. 43) originates from a midline raphe on the dorsal edge of the neck, passes ventrad, and inserts into a ventral median raphe. In *C*. *caretta*, the Pars intermandibularis (No. 42) and Pars oralis (No. 43) are separated ventrally, whereas in *L*. *kempii* they are completely continuous (Fig. 11A). Below the hyoid apparatus, in both species, some fibres of Pars oralis (No. 43) appear to attach to the ventral side of cornubranchial-I. Posteriorly, Pars oralis (No. 43) forms a continuous structure with the Pars spinalis (No. 41) in *L*. *kempii*, whereas only a few fibres connect these structures (No. 41, 43) in *C*. *caretta*.

### M. Depressor Mandibulae (No. 45)

The m. depressor mandibulae (No. 45) is a large fleshy muscle located at the posterolateral corner of the skull and is innervated by n. facialis (VII) ([126], [143] and [141]: table II/1-2) described the muscle as originating on the posterolateral face of the squamosal and the posterior process of the quadrate with a tendinous insertion onto the retroarticular process, as well as onto the ventral and lateral faces of the articular and its posteroventral tip.

**Figure 12:**
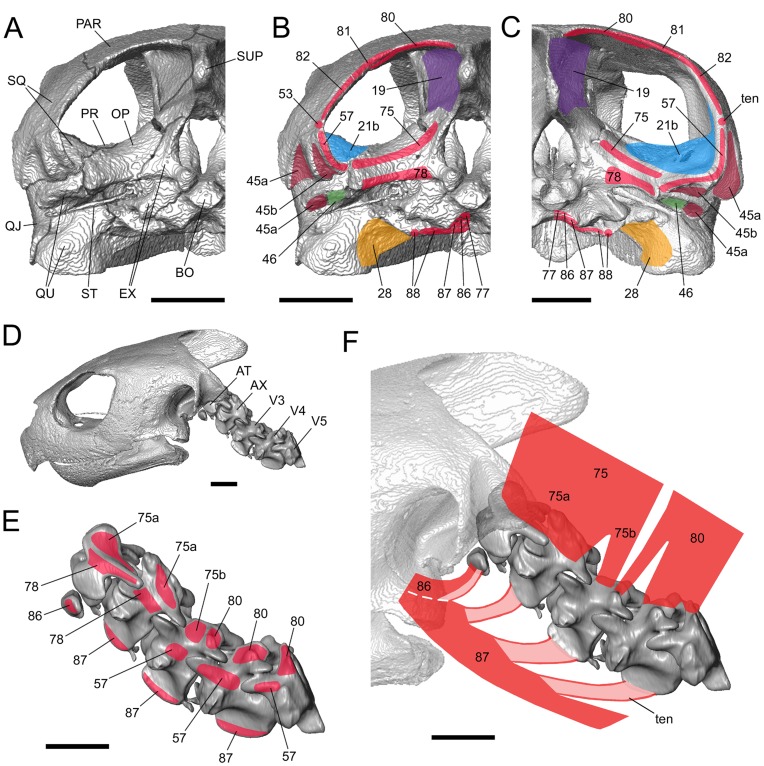
Neck muscle origins and insertions of two sea turtles illustrated using computer models based on micro-CT data. (**A** and **B**) *Caretta caretta* (XT757/07) and (**C**, **D**, **E** and **F**) *Lepidochelys kempii* (M009/08). Posterior views of the cranium showing (**A**) the osteology and (**B** and **C**) sites of muscle insertion. Lateral views of the neck (**D**) with skull, (**E**) showing sites of origin for muscles 75, 80, 86 amd 87, and (**F**) showing the structure of muscles 75, 80, 86 and 87. 19, m. adductor mandibulae externus Pars profundus; 21b, m. adductor mandibulae externus Pars superficialis posterior head; 28b, m. adductor mandibulae internus Pars pterygoideus small lateral head; 45a, m. depressor mandibulae lateral part; 45b, m. depressormandibulae medial part; 46, m. dilator tubae; 53, m. squamosobranchiale; 57, m. collosquamosus; 75, m. atlantoepistropheooccipitis; 77, m. atlantooccipitis; 80, m. collooccipitalis; 81, m. testocapitis; 82, m. testooccipitis; 86, m. longus colli Partes capitis-I et Pars capitis-II; 87, m. longus colli Partes capitis-I et Pars capitis-III; 88, m. retrahens capiti collique Pars carapacobasiooccipitalis; AT, atlas; AX, axis; BO, basioccipital; EX, exoccpital; OP, opisthotic; PAR, parietal; PR, prootic; QJ, quadratojugal; QU, quadrate; SQ, squamosal; ST, stapes; SUP, supraoccipital; ten, tendon; V3, third vertebra; V4, fourth vertebra; V5, fifth vertebra. Scale bars = 10 mm.

The muscle is composed of lateral (No. 45a) and medial (No. 45b) parts divided by an internal tendinous divison but both components merge prior to their insertion (Fig. 10D). The lateral part (No. 45a) originates from behind the cavum tympanicum on the posterolateral surface of the squamosal within a large ventrolateral depression, which has an obvious dorsal boundary, but shorter and deeper fibres also originate laterally from the posteroventral surface of the quadrate (Figs. 7A, 12B). The medial part (No. 45b) originates primarily from a depression in the posterior surface of the squamosal (Fig. 7A, 12B). The muscle parts converge approximately two thirds along the length of the complete muscle. The whole muscle inserts on the posterolateral corner of the short retroarticular process. The most medial region has a superficial tendon at the point of insertion that is continuous with that of the m. dilatator tubae (No. 46) (Fig. 10D). The shape of the muscle is similar in both species, but in *L*. *kempii* the lateral part (No. 45a) also originates from the ventral and lateral faces of the squamosal (Video S3) and has a tendinous attachment in its lateralmost region.

### M. dilatator Tubae (No. 46)

This muscle is innervated by n. facialis (VII) and has a close association with the m. depressor mandibulae (No. 45; [53], [143], [148]). The thin muscle originates by connective tissue from the edge of the quadrate just behind the eustachian tube and passes ventrally (Fig. 10D). Its mid point is connected anterolaterally to the posterior lining of the eustachian tube. Posterior to the articular, the muscle separates into two heads. The posterior head (No. 46a) inserts in the posteromedial tip of the prearticular, anterior to the medial part of the m. depressor mandibulae (No. 45). The anterior head (No. 46b) has a tendinous component and overlaps Pars pterygoideus ventralis (No. 28) of the internal adductor and inserts into the medial face of the prearticular, posteroventral to the insertion of the Pars pterygoideus dorsalis (No. 26) (Fig. 9G).

### M. branchiomandibularis Visceralis (No. 47)

This muscle is innervated by n. glossopharyngeus (IX) [143]. It originates from the dorsal ends of cornu branchial-I (Fig. 11B) and inserts with two heads. The first head (No. 47a) inserts onto the posteromedial aspect of the articular, whereas the second larger head (No. 47b) inserts on the complete medial aspect of the articular with a superficially tendinous component. In *L*. *kempii*, this muscle also originates from cornu branchial-I but via less substantial connective tissue. It inserts on the lower jaw around and beneath the m. depressor mandibulae (No. 45) and is partly fused with the m. constrictor colli complex (No. 41–43).

### M. plastrocapitis (No. 52)

This muscle is innervated by n. accessorius (XI). As we only had access to head- and anterior neck material, the origin of this strap-like muscle could not be identified. However, in both species, the origin is caudal to the sixth cervical vertebrae and presumably on the plastron [143]. In *C*. *caretta*, the muscle extends rostrad and inserts laterally, via a tendon, on the atlas and directly into connective tissue around the m. depressor mandibulae (No. 45) and cornu brachial-I. In *L*. *kempii*, the lateral aspect of the muscle is partly fused with the m. constrictor colli complex (No. 41–43) along its course and the insertion onto the atlas is not tendinous.

### M. squamosobranchiale (No. 53)

This muscle is innervated by n. accessorius (XI) and originates from the posterior end of the squamosal above the origin of m. depressor mandibulae (No. 45) and the insertion of m. collosquamosus (No. 57). M. squamosobranchiale inserts onto the distal edge of cornu branchial-I (Fig. 11B). The muscular unit m. squamosobranchiale (No. 53) appears to be absent in *L*. *kempii*, but in its place is a very thin tendinous structure between the cornu branchial-I and the squamosal.

### M. branchiohyoideus (Principalis) (No. 55)

This muscle is innervated by n. hypoglossus (XII). In both taxa the muscles originates from the anterior edge of cornu branchial-I, ventral to the origin of m. branchiomandibularis visceralis (No. 47) and passes anteriorly to insert onto the corpus hyoidei (Fig. 10A).

### M. collosquamosus (No. 57)

This muscle is innervated by n. hypoglossus (XII). It originates from cervical vertebrae-3 and -4, and possibly also from -5. It passes rostrolaterad and fans out at the level of the atlas to insert on the posterior surface of the squamosal, ventromedial to the insertion of m. squamosobranchiale (No. 53), dorsomedial to m. depressor mandibulae (No. 45), and lateral to m. atlantoepistropheooccipitis (No. 75) (Fig. 11G and 12B, C). The ventral part of the insertional area of the m. collosquamosus (No. 57) includes the region where the posterior squamosal spine is found in large specimens of *C*. *caretta* (e.g. LDUCZ×11; UMZC R 4606). The insertion of the m. collosquamosus (No. 57) onto the back of the skull appears to be broader in *L*. *kempii* (and no spine is present).

### M. coracohyoideus (Pars Principalis et Pars Interbranchialis) (No. 58+60)

This muscle is innervated by n. hypoglossus (XII) [143]. Problematically, some or all of it has previously been referrred to as the “m. rectus cervicis” (e.g. [128], [130] and see [143]). The main unit (No. 58) reportedly originates from the pectoral girdle [143] and inserts on the ventral surface of cornu branchial-II as well as on the posterior edge of the proximal half of cornu branchial-I (Figs. 11A, B). A small unit (No. 60) connects the ventral surface of cornu branchial-II to the posterior edge of the proximal half of cornu branchial-I (Fig.11B).

### The Tongue

The tongue of turtles is composed of several small muscular units including, for example, the mm. genioglossus (No. 63) et hypoglossus (No. 69) [143]. We did not investigate the individual structure of these, only the gross morphology of the tongue as a whole. The tongue is located in front of the pharynx, supported by the hyoid apparatus, and occupies the space between the occlusal surfaces of the lower jaws. In both taxa it is fleshy with a slightly wrinkled dorsal surface (Figs. 11C–E).

### M. atlantoepistropheooccipitis (No. 75)

This muscle is innervated by the dorsal branch of the cervical spinal nerve-1 and includes what has been referred to problematically as the rectus capitis superficialis (e.g. [128]; see [143]). The m. atlantoepistropheooccipitis (No. 75) is located above the m. constrictor colli (No. 41–43) and originates by two heads, one (No. 75a) from the dorsolateral surfaces of the first two cervical vertebrae and the other (No. 75b) as a small slip from the anterodorsal surface of the third cervical vertebrae (Figs. 11F, G and 12E, F). Both heads merge shortly after origin, and the muscle fans out towards the braincase inserting on the posterior surface of the squamosal, opisthotic, and exoccipital, above the insertion of m. atlantooccipitalis (No. 78), and medial to the insertion of m. collosquamosus (No. 57) (Figs. 12B, C). In *C*. *caretta*, the insertion may also involve the posterior edge of the supraoccipital (Figs. 11F, 12B), whereas in *L*. *kempii* the insertion is less extensive medially and ends at or near the suture between the opisthotic and the supraoccipital (Figs. 11I, 12C).

### M. atlantooccipitis (No. 77)

This muscle is innervated by the dorsal branches of cervical nerves [143]. It originates from the lateral face of the atlas and inserts via a tendon on the medialmost part of the ventral surface of the basioccipital (Fig. 12B, C), posterior to the insertions of mm. longus colli (No. 86+87) et carapacobasiooccipitalis (No. 88).

### M. atlantoopisthoticus (Principalis) (No. 78)

This muscle is innervated by the dorsal branches of cervical nerves [143]. It originates dorsolaterally from the atlas and axis (Fig. 12E), extends ventrad, and inserts on the posterior face of the opisthotic and the exoccipital (Fig. 12B, C).

### M. collooccipitalis (No. 80)

This muscle is related to the “m. obliquus capitis” of Shah [129] (but see [143]), and originates by three separate strap-like muscle heads from the connective tissue over the neural spines of the neck vertebrae: two from above the fourth and the fifth cervical vertebrae and a more slender contribution from above the third (Figs. 11G and 12F). It inserts onto connective tissue dorsal to and between the m. adductor mandibulae externus Partes medialis (No. 17) et profundus (No. 19) and also into the posterior edge of the parietal and supraoccipital (Fig. 12B, C).

### Mm. Testocapitis et Testooccipitis (No. 81+82)

These are the most superficial muscles of the neck, innervated by dorsal branches of cervical nerves. In *C*. *caretta* and *L*. *kempii*, the muscles are more or less fused anteriorly and therefore need to be considered as one muscle *sensu stricto* herein [143]. The muscle usually originates from the anterior edge of the carapace (e.g. [128], [130]). In our specimens of *C*. *caretta* and *L*. *kempii*, the Mm. testocapitis et testooccipitis (No. 81+82) inserts on the posterior edge of the squamosal so that its mediolateral edge overlies the lateral edge of the m. collooccipitalis (No. 80) (Figs. 12B, C). The lateral end of its insertion is located dorsal to the insertion point of m. squamosobranchiale (No. 53).

### M. transversalis Cervicis (No. 83)

This muscle originates from the ventrolateral surfaces of the fifth to the seventh vertebrae and passes rostrad along the side of the neck before converging and inserting anteriorly in the region of the atlas and axis.

### M. longus Colli Partes Capitis-I et Pars Capitis-II/III (No. 86 et No. 87)

The anterior unit (No. 86) has a mainly fleshy origin from the ventral surface of the atlas. The posterior unit (No. 87) is tendinous (Fig. 11H, and 12E, F) and arises from the ventral keels of the second to the fourth cervical vertebrae (Figs. 11C, and 12E, F). The two units fuse shortly after their origin, run rostrad and insert on the ventral surface of the basioccpital (Figs. 11H and 12B, C) with the anteriormost fibres (No. 86) inserting medial to the posterior fibres (No. 87), and anterior to the insertion of m. atlantooccipitalis (No. 77).

### M. retrahens Capiti Collique Pars Carapacobasioccipitalis (No. 88)

As a part of the multiportioned m. retrahens collique, this unit (No. 88) originates from the ventral face of the carapace [143] and passes along the side of m. longus colli (No. 87). At the level of the third cervical vertebra it (No. 88) becomes entirely tendinous and inserts on the basioccipital tubercle posterolateral to the insertion of the m. longus colli (No. 86+87; Fig. 11G, H).

## Discussion

### Sea Turtle Muscle Anatomy

*Caretta caretta* and *Lepidochelys kempii* share a number of general features related to their musculature. However, available descriptions of other taxa suggest that many of these similarities are more widely distributed within turtles [44], [76], [126], [136], [143], [149]–[152]. An origin of the m. adductor mandibulae externus Pars medialis (No. 17) from the rostral face of the quadrate is also found in *Eretmochelys imbricata* [76], whereas an origin of the m. adductor mandibulae internus Pars pterygoideus dorsalis (No. 26) that includes the palatine appears to be present in all cheloniids, many other cryptodires and some chelids (e.g. [44], [126], [136]). There is one character that appears to be a synapomorphy of *C*. *caretta* and *L*. *kempii*: insertion of the m. adductor mandibulae internus Pars pseudotemporalis principalis (No. 23) into a Zwischensehne and, in turn, the m. intramandibularis (No. 25). Whether this character is present in *L*. *olivacea* remains to be determined but it potentially provides a character for diagnosing the Carettini.

Despite a general similarity in the cranial muscle anatomy of *C*. *caretta* and *L*. *kempii* there are also several differences. These include the relative proportions of different muscle parts, the exact location of origins and insertions, and the relationship between muscles and particular aponeuroses. In *L*. *kempii,* for example, the second head of the m. adductor medialis externus Pars superficialis (No. 21b) has a relatively more extensive origin than in *C*. *caretta* (Fig. 7D, 12B), whereas in *C*. *caretta*, the lateralmost part of the muscle (No. 21c) has a more ventrally located insertion than in *L*. *kempii* (Fig. 12). In *L*. *kempii* the m. depressor mandibulae (No. 45) originates from more lateral and ventral locations on the squamosal, than in *C*. *caretta*. In general muscle organisation tends to be more complicated in *C*. *caretta* than in *L*. *kempii* where the m. adductor medialis externus (No. 17–21) and m. constrictior colli complex (No. 41–43) are less finely divided, the m. branchiomandibularis visceralis (No. 47) is less distinct, and the m. squamosobranchiale (No. 53) appears to be entirely absent. Whether this is related to the greater adult size of *C*. *caretta* remains to be tested.

One feature apparently common to Chelonioidea appears to be the presence of a large fleshy m. adductor mandibulae Pars superficialis (No. 21) with a broad origin inside the cheek and an insertion on the lateral side of the lower jaw [44], [76], [126], [136], [152]. Chelonioidea all lack m. adductor mandibulae externus Pars medialis inferior (No. 18), m. zygomaticomandibularis (No. 22), and m. adductor mandibulae externus Pars profundus atypica (No. 20) [76], [126], [136], and seem to share a number of other muscle characters (see Table 4).

**Table 4:** Muscle features common to Chelonioidea. Compiled from Lakjer [76], Gnanamuthu [138]; Poglayen-Neuwall [136], [141], Schumacher [126], [141], [142], [149]–[151], Werneburg [44], [143], [152].

No.	Character
1	Origin of the m. adductor mandibulae externus Pars medialis (No. 17) includes the anterior surface of the quadrate but not the capsule of the jaw joint, postorbital, prootic, supraccipital, or parietal.
2	Origin of the m. adductor mandibulae externus Pars profundus (No. 19) extends more posteriorly than that of the Pars superficialis.
3	Origin of the m. adductor mandibulae externus Pars profundus (No. 19) includes the supraoccipital but not the postorbital, opisthotic, or prootic.
4	Origin of the m. adductor mandibulae Pars superficialis (no. 21) includes the quadratojugal, squamosal, postorbital, and jugal.
5	Insertion of the m. adductor mandibulae internus Pars pterygoideus dorsalis (no. 26) includes the prearticular.
6	Origin of the m. adductor mandibulae internus Pars pterygoideus ventralis (No. 28) includes the palatine.
7	Origin of the m. depressor mandibulae (No. 45) includes the posterior face but not the dorsal surface of the squamosal.

### Feeding Behaviour

Sea turtles generally begin life as pelagic carnivores (or at least omnivores) but as adults they demonstrate a range of feeding ecologies [2], [12], [23], [25], [97], [100], [118]. *Dermochelys coriacea* remains highly pelagic and forages throughout the water column for jellyfish and salps [25], [153]. *Caretta caretta* moves to coastal waters and feeds opportunistically on a variety of benthic prey (sea pens, crabs, and molluscs) with seasonal variations [25], [107]–[110]. *Chelonia mydas* also moves to near shore habitats but becomes herbivorous and feeds on sea grass and algae [25], [153]. *Lepidochelys kempii* tends to move to coastal waters as an adult when it generally preys on crustaceans such as crabs [25], [107], [119] but *L*. *olivacea* is more oceanic [25]. Adult *Natator depressus* generally inhabit coastal waters and shallow bays where they feed on soft corals, sea grass, sea pens, and soft-bodied invertebrates [154], whereas *E*. *imbricata* usually lives in nearshore reefs and eats sponges [155]. Despite this diversity of foraging mode, the feeding kinematics of sea turtles have not specifically been examined, but some inferences can be made based on the observed anatomy and known feeding behaviour in other aquatic turtles.

Feeding within an aquatic medium is problematic because a predatory strike can deflect prey away from the mouth [32], [127], [156]–[159]. Observations of freshwater turtles show that this problem is overcome by rapid expansion of the esophagus as the jaws open and retraction of the relatively large and well-ossified hyoid apparatus by the m. branchiohyoideus (No. 55). This creates a negative pressure within the buccopharyngeal cavity so that food is effectively sucked in [32], [127], [157], [158]. Freshwater turtles also use the neck to move the head rapidly towards prey [160] and often have a dorsoventrally compressed and streamlined skull to reduce water displacement [32], [161], [162].

The relatively small tongue in *C*. *caretta* and *L*. *kempii* is consistent with some use of suction feeding [32], [127], [156], but the generally shorter neck limits head mobility and the hyoid apparatus is not as well-developed. Moreover, in contrast to many freshwater turtles [32], [163], *C*. *caretta* and *L*. *kempii* possess a number of cranial features that suggest they are capable of forceful biting [23]. These include the wide trituration surfaces, the reinforced palate, and a large fused symphysis (Fig. 3; [19], [29]). They also possess a tall skull, a trait that is associated with a relatively greater biting performance [163], perhaps because its associated with muscles that are larger and have fibres with a more perpendicular orientation relative to the long axes of the jaws. A tall skull is also better shaped to resist the bending and torsional forces related to forceful biting [164]–[166]. Shape analysis of testudinid and emydid turtles found that small rather than large ventrolateral emarginations are associated with a durophagous diet [162]. Pitting on the bony surfaces that contact the rhamphothecae are probably related to nutrient supply but they may also serve to increase the surface area, and therefore the strength, of attachment. Both *C*. *caretta* and *L*. *kempii* tend to take rather slow moving but occasionally armoured prey such as sea pens, crabs, and molluscs [108], [109], [119]. Fish consumption is less common, and when recorded it probably represents feeding on the discarded by-catch of commercial fishing vessels [119], [167], [168]. Thus, for these turtles forceful biting once prey is caught may be more important than speed and suction feeding during prey acquisition.

The large jaw muscles are also consistent with forceful biting. Both *C*. *caretta* and *L*. *kempii* possess a coronar aponeurosis into which jaw muscles from the back of the adductor chamber insert (e.g. m. adductor mandibulae externus Pars profundus [No. 19]). Because this aponeurosis loops over the otic area (Fig. 2B) before inserting into the lower jaw its line of action is close to vertical regardless of how far back the origins of the most posterior jaw muscle portions lie. As a result of this pulley system and pennation, the volume of adductor muscle is large and has effective leverage [29], [150], [169]–[171]. The bony covering of the cheek is bowed outward, so there is nothing to suggest that the fully ossified temporal region restricts the volume of the large m. adductor mandibulae Pars superficialis which inserts on the lateral side of the lower jaw.

In both *C*. *caretta* and *L*. *kempii*, the articular surface on the lower jaw is at least twice the length of the quadrate condyle, and the articular surface bears two parallel troughs, one on either side of a median ridge (Fig. 4). This suggests some relative antero-posterior translation during jaw movement. The symmetrical shape of the articular surface is important in permitting such movement given that the lower jaw symphysis is immobile (see [172]). Based on muscle arrangements, this sort of “propalinal” movement has been considered to occur widely in turtles [126], [170] but the relationships between the articular and quadrate are poorly known and essentially undescribed in most taxa.

The orientations of muscle paths can provide a good indication of their role during jaw movement. For example, Curtis *et al*. [173] showed that muscle activity predicted by a multibody dynamics computer model according to muscle orientation is very similiar to that recorded *in vivo*. In the sea turtles described here, the posteriorly located m. depressor mandibulae opens the lower jaw, perhaps with some support from the most posterior fibres of the Pars pterygoideus ventralis (No. 28). Otherwise the orientation of the Partes pterygoidei (No. 26, 28) suggests their main function is to close the jaws and pull them forward, particularly when the jaws are wide open [51], [174]. The large m. adductor mandibulae externus Pars profundus (No. 19) and the vertically orientated m. adductor mandibulae externus Pars superficialis (No. 21) would provide the greatest power during the final stages of jaw closure [51], [174]. The origin and insertion of the m. adductor mandibulae externus Pars medialis (No. 17) are positioned so that the long axis of that portion is approximately parallel to that of the lower jaw when the jaws are closed. It should therefore be very effective at pulling the lower jaw posteriorly when the jaws are closed or almost closed. This suggests palinal rather than proal movement: the jaws moving posteriorly rather than anteriorly at jaw closure [169], [174], [175]. Whether this movement serves to enhance food prehension or food reduction remains uncertain. In addition to allowing palinal jaw movement, the counter orientation of the internal and external adductor portions may reduce reaction forces at the jaw joint [127], [147].

The ligamentum quadratomaxillare [76], [136], [143], [149], [151] may represent a passive tension cord (cf. [176]) for resisting tensile strains that might arise along the ventrolateral edge of the dome-like cranium during biting. This hypothesis may be tested using finite element modelling similar to that used in Curtis *et al*. [177].

The skull mechanics of *Ch*. *mydas*, *E*. *imbricata*, *L*. *olivacea*, and *Natator depressus* are probably broadly comparable to those of *C*. *caretta* and *L*. *kempii*, but this requires further examination with appropriate consideration for ontogenetic and geographic variation [121], [178]. *Dermochelys coriacea* differs from other chelonioids [142] in both skull shape and muscle arrangement. The skull is relatively tall with a short crista supraoccipitalis [179], a cartilago transiliens is absent, and the main jaw adductor muscles do not loop over the ear region [76], [140], [142] but have a more direct path. The implications of this derived but apparently simpler arrangement require further investigation.

### Muscles and Skull Emargination

Sea turtle skulls are of particular interest to comparative anatomists because, unlike those of most extant turtles, which have variably developed ventrolateral and posterodorsal emarginations, their temporal region is almost entirely covered by bone (Figs. 3, 14; [8], [29], [100], [124]). The postorbital, jugal, quadratojugal, parietal, and squamosal all contribute to the temporal region and the parietal, squamosal, and postorbitals all meet one another. Ventrolateral and posterodorsal emarginations are present but small (reviewed by Zdansky [27], Kilias [28], Gaffney [29], and Werneburg [122]). This means that, unlike the skulls of most reptiles which are composed of a rod-like framework of bone [177], [180], [181], the skulls of sea turtles have a shell- or dome-like shape. Although the detailed arrangement of individual bones differs, this condition is superficially comparable to that of stem turtles (e.g. *Proganochelys quenstedti*, [182]) and to the anapsid skull condition found in many extinct non-amniotes, parareptiles, and basal eureptiles (e.g. *Captorhinus* sp., [183]).

**Figure 13:**
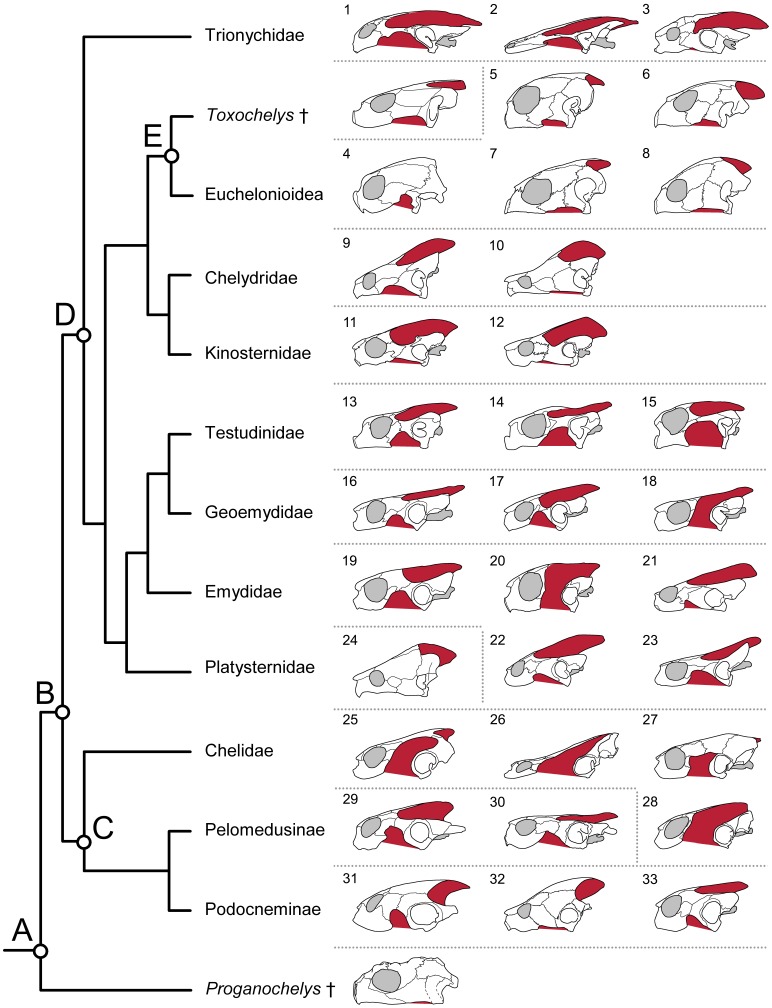
Turtle skulls plotted against the topology of Shaffer (2009) with the addition of *Proganochelys* and *Toxochelys*. Major phylogenetic groupings include (A) Testudinata, (B) Testudines, (C) Pleurodira, (D) Cryptodira, and (E) Chelonioidea. Euchelonioidae is used in the sense of Gaffney and Meylan [202]. Individual skulls represent (**1**) *Lissemys punctata*, (**2**) *Chitra indica*, (**3**) *Carrettochelys insculpta*, (**4**) *Dermochelys coracea*, (**5**) *Chelonia mydas*, (**6**) *Lepidochelys kempii*, (**7**) *Eretmochelys imbricata*, (**8**) *Caretta caretta*, (**9**) *Chelydra serpentia*, (**10**) *Macrochelys temmincki*, (**11**) *Kinosternon subrubrum*, (**12**) *Sternotherus odoratus*, (**13**) *Stigmochelys* ( = *Testudo*) *pardalis*, (**14**) *Kinixys belliana*, (**15**) *Gopherus polyphemus*, (**16**) *Cuora amboinensis*, (**17**) *Melanochelys* ( = *Nicoria*) *trijuga*, (**18**) *Heosemys* ( = *Geoemyda*) *spinosa*, (**19**) *Clemmys caspica*, (**20**) *Terrapene ornata*, (**21**) *Graptemys geographica*, (**22**) *Malaclemys terrapin littoralis*, (**23**) *Emys blandingii*, (**24**) *Platysternon megacephalum*, (**25**) *Emydura* sp., (**26**) *Chelodina expansa*, (**27**) *Pseudemydura umbrina*, (**28**) *Chelodina novaeguinae*, (**29**) *Pelusios sinuatus*, (**30**) *Pelusios niger*, (**31**) *Podocnemis expansa*, (**32**) *Peltocphalus dumeriliana*, and (**33**) *Pseudemys floridana*. Skulls re-drawn from Zangerl [30], Gaffney [29], [31], [177], and Matzke [22]. Taxonomy follows Fritz and Havaš [24]. Grey areas indicate orbits, braincase or naris whereas dark red indicates skull portions within an emargination. Note that the closest skull to the group name does not necessarily indicate the plesiomorphic condition for that group.

Although skull emarginations are widespread in extant non-chelonioid turtles, there is substantial variation as to their size and shape [29], [30], [122]. The posterodorsal emarginations are largest in most turtles (e.g. Fig. 13.1–3, 9–12, 21, 29, 31–33), but the ventrolateral ones are larger in chelids (e.g. Fig. 13.25, 27). Both emarginations tend to be large in trionychids and testudinids, resulting in a superficially diapsid-like skull lacking infra- and posttemporal arcades (e.g. *Gopherus polyphemus*, Figs. 13.1, 13–15), and this is taken one step further in some geoemydids (*Hieremys annandalii*, *Heosemys spinosa*, Figs. 13.18), emydids (e.g. *Terrapene carolina* and *T*. *ornata*, Fig. 13.20), and chelids (e.g. *Chelodina expansa*, *Chelodina novaeguinae*, Figs. 13.26,28) where the emarginations are confluent. By contrast, *Carettochelys insculpta* (Fig. 13.3), *Macrochelys temminckii* (Fig. 13.3), kinosternids (e.g. *Kinosternon subrubrum*, *Sternotherus odoratus* Fig. 13.11,12), and some podocnemids (e.g. *Peltocephalus dumeriliana*, Figs. 13.32) almost lack a ventrolateral emargination. The same is true for *Platysternon megacephalum*, arguably the least emarginated turtle outside Chelonioidea, but this species is unusual in having a small jugal (Fig. 13.24).

The near absence of emargination in extant sea turtles appears to be secondary, because they all have living or extinct relatives with moderate or large emarginations. Early fossil Chelonioidea such as *Toxochelys latiremis* (Fig. 13) and *Allopleuron hoffmani*, and early cryptodires (e.g. *Ordosemys* sp., [39]) possess an emarginated temporal region comparable to that of many extant non-chelonioid taxa [22], [29], [36]. Within pleurodires, *Peltocephalus dumeriliana* and *Erymnochelys madagascariensis* are nested amongst more emarginated extant members of Podocneminae and related fossil taxa [43], [184], [185]. Similarly, *Pseudemydura umbrina* is phylogenetically nested amongst highly emarginated chelids [43] and, like other clade members, lacks a quadratojugal [29], [31]. Therefore, secondary enclosure of the temporal region appears to have occurred within testudines several times.

Many stem turtles essentially lack posterodorsal emarginations (e.g. *Odontochelys semitestacea*, Late Triassic, China: [186]; *Proganochelys quenstedti*, Late Triassic, Germany: [182]; *Kayentachelys aprix*, Early Jurassic, USA: [187]; *Condorchelys antiqua*, Middle Jurassic, Argentina: [188]), and ventrolateral emarginations, if present, are small (e.g. *O*. *semitestacea*, [186]: 497; *P*. *quenstedti* [pers. obs. MEHJ and IW]; *K*. *aprix*, [187]). Therefore, the plesiomorphic condition for Testudinata (Testudines+stem group taxa) it is not necessarily the same as for the less inclusive Testudines.

With a computer model of the extant rhynchocephalian *Sphenodon* as an example taxon, Curtis *et al*. [177] recently used Finite Element Modelling to show that when all loading conditions are taken into account, all of the bone in a reptile skull contributes to its structural integrity. This is consistent with the theory that skull shape is strongly linked to mechanical loading or strain (e.g. [48]–[52], [54], [56], [122], [176], [181]): from birth, as the skull grows around the sensory organs, loading from the muscles and feeding apparatus iteratively modify the shape of the skull via remodelling [189], [190]. Correspondingly, it is known that aberrant loading conditions lead to aberrant skull shapes (e.g. [53], [55], [191]).

Given the apparent association between loading, muscles, and skull shape, the relationship between the m. adductor mandibulae externus Pars superficialis (No. 21) and the medial wall of the temporal region in turtles warrants consideration. In chelonioids, the muscle’s fleshy origin should mean that resulting loading is evenly spread rather than locally concentrated at a tendinous origin, a condition that may promote the deposition of bone [54]. A similar muscle arrangement is found in the non-chelonioid cryptodire *Kinosternon subrubrum* [29], [136], which has a large jugal and a small ventrolateral emargination, but is absent in several turtles with large ventrolateral emarginations (e.g. *Lissemys punctata*, *Terrapene carolina*, *Testudo graeca*, *Pelusios subniger*, and chelids such as *Emydura macquarii* and *Chelus fimbriatus*; [29], [32], [136], [149]–[151], [192]). However, in the non-chelonioids *Sternotherus odoratus* and *Platysternon megacephalum* [136], [149]–[151] that lack ventrolateral emarginations, a broad fleshy origin of the m. adductor mandibulae externus Pars superficialis (No. 21) (including the jugal surface) is apparently not present. Thus, although some aspects of the pattern are suggestive of a correspondence between muscle anatomy and skull morphology, the relationship is not clearcut, and the presence or absence of emarginations probably involves a combination of factors [44], [51], [52], [122], [189]. The rhynchocephalian lepidosaur *Sphenodon* also has a large superficial adductor muscle, but it arises from the fascia that attaches to the edges of the large lower temporal fenestra so that loading is concentrated at the margins with bone deposition limited to this edge [147], [177].

The relatively small posterodorsal emargination in chelonioids may be associated with the more posterior origin of m. depressor mandibulae from the squamosal [76], [126], [138], [142]. Only in *L*. *kempii* does the m. depressor mandibulae encroach on the lateral surface of the squamosal, and this taxon has wider posterodorsal emarginations and a more dorsolateral origin of the m. adductor mandibulae externus Pars superficialis from the squamosal. Non-chelonioid turtles with a more posterior origin of m. depressor mandibulae include *Chelydra serpentina*, *Emys orbicularis*, *Malaclemys terrapin litoralis,* and *Podocnemis expansa* [136], [137], [146], [193], [194]. Whereas the posterodorsal emarginations in *P*. *expansa* are small [29], those in *Chely*. *serpentina*, *Em*. *orbicularis* and *M*. *terrapin litoralis* are relatively large [29], [123]. Also, although most turtles with extensive posterodorsal emarginations (e.g. *Terrapene carolina*, *Pelodiscus sinensis*, *Lissemys punctata* [136], [148], [192]) have a laterally located origin of m. depressor mandibulae (No. 45) [44], [143], so do some turtles with little or no posterodorsal emargination (e.g. chelids such as *Emydura macquarii* [193], [194]).

Of all chelonioids, *D*. *coriacea* arguably shows the least emargination (Fig. 13.4), but this taxon is also unusual in lacking the trochlearis system of the adductor musculature and in having a more linear orientation of the external adductor portions [137], [142]. In addition, the internal adductor musculature does not originate from the processus descendens parietalis, an arrangement secondarily in this taxon lost after it appeared on the stem line of Testudines [171]. The absence of both a pulley system and a posterodorsal emargination in *D*. *coriacea* supports the hypothesis that these two characters are functionally correlated [171], [122].

Further anatomical and modelling work on these and other reptiles may shed light on the relationship between soft and hard tissues in the generation of skull form. However, detailed anatomical surveys are an essential foundation for this work.

## Methods

### Materials

Between 2007 and 2008 three *Caretta caretta* and two *Lepidochelys kempii* specimens were recovered *post mortem* by the UK Cetacean Strandings Investigation Programme from beaches in Wales and Scotland (United Kingdom) (Table 2). They were identified by external examination and measured. All the specimens in our study represent immature animals [195] and thus individuals at the oceanic feeding stage of their life history [118]. Prior to our dissections, specimens XT161/08 and XT043/08 had their skull roofing bones cut open and brains removed for pathological examination.

Osteological material was also examined from collections at and the Natural History Museum of Los Angeles County, USA (LACM); Grant Museum of Zoology, University College London, UK (LDUCZ); and University Museum of Zoology Cambridge, UK (UMZC) (Table 1).

### Dissections

Material was received and stored as frozen, but prior to dissection it was soaked overnight in diluted commercially available fabric softener. This made the tissues much easier to separate [196]. Dissection was carried out and documented using a Canon 8 Mega Pixel Digital Camera with macro function. A Wild stereobinocular microscope was also used to study structures in detail. Innervations were largely determined from the literature and previous dissections [143].

### Micro-Computed Tomography

One specimen each of *L*. *kempii* (M009/08) and *C*. *caretta* (XT757/07) were were subjected to micro-computed tomography (micro-CT) at the University of Hull, UK using a X-Tek HMX 160 scanner using a Beryllium target and aperture of 75%. To reduce beam hardening, the X-rays were filtered through a 0.1 copper plate. M009/08 was scanned using 1149 projections averaging 64 frames per projection, whereas XT757/07 was scanned using 1113 projections averaging 32 frames per projection. Anatomical structures were segmented using the software Amira 4.1 (Konrad-Zuse-Zentrum für Informationstechnik Berlin). Voxel resolution was 0.121 mm^3^ for M009/08 and 0.081 mm^3^ for XT757/07. The scan of *L*. *kempii* (M009/08) was particularly successful and included details of the muscle arrangement (Fig. 3) despite the absence of iodine staining as described by Metscher [197] and Jeffery *et al*. [198] (but see also [199]).

### Terminology

The names used for neuro- and dermatocranial elements of turtles mainly correspond to those of Gaffney [29], [123], whereas the names used for splanchnocranial elements follow Fürbringer ([200]: hyal apparatus). Anatomical terms for the cervical vertebrae are similar to those used by Herrel *et al*. [130] and the nomenclature of cranial musculature and other soft tissues follows Werneburg [143] (Table 3). Taxonomic nomenclature follows Fritz and Havaš [24] and Joyce *et al*. [201].

### Note Added Post- Acceptance

A recent publication on the ontogenetic scaling of bite force in *Caretta caretta* confirms that these turtles are durophagus as adults [203].

## Supporting Information

Video S1 **Cranium of of a young** ***Caretta caretta*** **(XT757/07) based on reconstructions from micro-CT data.** The surface model has been simplified to 410000 faces. Also see figure 2.
(MPEG)

Video S2 **Cranium of of a young** ***Lepidochelys kempii*** **(M009/08) based on reconstructions from micro-CT data.** The surface model has been simplified to 500000 faces. Also see figure 2.
(MPEG)

Video S3 **Skull of a young** ***Lepidochelys kempii*** **(M009/08) with parts of the right temporal region absent and jaw muscles shown.** The model is based on reconstructions from micro-CT data. Also see figures 3 and 8.
(MPEG)
